# Novel Staphylococcal Glycosyltransferases SdgA and SdgB Mediate Immunogenicity and Protection of Virulence-Associated Cell Wall Proteins

**DOI:** 10.1371/journal.ppat.1003653

**Published:** 2013-10-10

**Authors:** Wouter L. W. Hazenbos, Kimberly K. Kajihara, Richard Vandlen, J. Hiroshi Morisaki, Sophie M. Lehar, Mark J. Kwakkenbos, Tim Beaumont, Arjen Q. Bakker, Qui Phung, Lee R. Swem, Satish Ramakrishnan, Janice Kim, Min Xu, Ishita M. Shah, Binh An Diep, Tao Sai, Andrew Sebrell, Yana Khalfin, Angela Oh, Chris Koth, S. Jack Lin, Byoung-Chul Lee, Magnus Strandh, Klaus Koefoed, Peter S. Andersen, Hergen Spits, Eric J. Brown, Man-Wah Tan, Sanjeev Mariathasan

**Affiliations:** 1 Dept. of Infectious Diseases, Genentech, Inc., San Francisco, California, United States of America; 2 Protein Chemistry, Genentech, Inc., San Francisco, California, United States of America; 3 AIMM Therapeutics and Department of Cell Biology and Histology, Academic Medical Center, Amsterdam, The Netherlands; 4 Proteomics Lab, Genentech Inc., San Francisco, California, United States of America; 5 Translational Immunology, Genentech, Inc., San Francisco, California, United States of America; 6 Division of Infectious Diseases, University of California, San Francisco, California, United States of America; 7 Antibody Engineering, Genentech, Inc., San Francisco, California, United States of America; 8 Biochemical and Cellular Pharmacology, Genentech, Inc., San Francisco, California, United States of America; 9 Structural Biology, Genentech, Inc., San Francisco, California, United States of America; 10 Early Discovery Biochemistry, Genentech, Inc., San Francisco, California, United States of America; 11 Symphogen A/S, Lyngby, Denmark; Vanderbilt University, United States of America

## Abstract

Infection of host tissues by *Staphylococcus aureus* and *S. epidermidis* requires an unusual family of staphylococcal adhesive proteins that contain long stretches of serine-aspartate dipeptide-repeats (SDR). The prototype member of this family is clumping factor A (ClfA), a key virulence factor that mediates adhesion to host tissues by binding to extracellular matrix proteins such as fibrinogen. However, the biological siginificance of the SDR-domain and its implication for pathogenesis remain poorly understood. Here, we identified two novel bacterial glycosyltransferases, SdgA and SdgB, which modify all SDR-proteins in these two bacterial species. Genetic and biochemical data demonstrated that these two glycosyltransferases directly bind and covalently link N-acetylglucosamine (GlcNAc) moieties to the SDR-domain in a step-wise manner, with SdgB appending the sugar residues proximal to the target Ser-Asp repeats, followed by additional modification by SdgA. GlcNAc-modification of SDR-proteins by SdgB creates an immunodominant epitope for highly opsonic human antibodies, which represent up to 1% of total human IgG. Deletion of these glycosyltransferases renders SDR-proteins vulnerable to proteolysis by human neutrophil-derived cathepsin G. Thus, SdgA and SdgB glycosylate staphylococcal SDR-proteins, which protects them against host proteolytic activity, and yet generates major eptopes for the human anti-staphylococcal antibody response, which may represent an ongoing competition between host and pathogen.

## Author Summary

*Staphylococcus aureus* and *S. epidermidis* are major bacterial pathogens that can cause life-threatening human diseases. Following entry into the circulation, *S.aureus* can infect virtually any organ. However, it must first counter antibacterial mechanisms of the innate immune system, including those involving macrophages and neutrophils. Important for staphylococcal adhesion to and successful colonization of host tissues, is a family of bacterial surface proteins containing multiple repeats of serine-aspartate repeats (SDR) adjacent to an adhesive A-domain. The biological functions of the SDR-domain of these SDR proteins remain elusive. We found that the SDR-domain of all staphylococcal SDR proteins is heavily glycosylated. We identified two novel glycosylases, SdgA and SdgB, which are responsible for glycosylation in two steps, and found that this glycosylation protects the adhesive SDR proteins against proteolytic attack by human neutrophil cathepin G. Since pathogen binding to human tissues, including the extracellular matrix protein fibrinogen, depends on SDR proteins, this glycosylation may be important for successful colonization of the human host. We also show that the SdgB-mediated glycosylation creates an immunodominant epitope for highly opsonic antibodies in humans. These antibodies account for a significant proportion of the total anti-staphylococcal IgG response.

## Introduction

*Staphylococcus aureus* and *S. epidermidis* are successful human commensals that primarily colonize the nares and skin. *S. aureus* can also invade a variety of tissues, leading to life-threatening infections. Recently emerged strains of *S. aureus* show increased virulence and enhanced ability to cause disease in otherwise healthy individuals. In addition, the recent development of resistance to antibiotics, in particular methicillin, have made *S. aureus* infections more difficult to treat. Currently, the most prevalent and most virulent clinical strain of methicillin resistant *S. aureus* (MRSA) is USA300, which has the capacity to produce a large number of virulence factors and cause mortality in infected individuals [1]. *S. epidermidis*, which is closely related to *S. aureus*, is often associated with hospital-acquired infections, and represents the most common source of infections on indwelling medical devices.

The factors mediating colonization of human tissues by *S. aureus* and *S. epidermidis* are complex and not yet fully elucidated, but have been studied in many animal models of *S. aureus* infection. Tissue colonization involves interactions of several *S. aureus* surface proteins with host cells and extracellular matrix. Using *in vitro* models, several *S. aureus* surface proteins, including clumping factor (Clf)A and ClfB, are important for adherence to mammalian cell lines and purified extracellular matrix proteins [2]. In addition, it is believed that ClfA is a key factor in triggering sepsis [3]. ClfA and ClfB are members of a family of cell wall proteins, characterized by a large stretch of serine-aspartate dipeptide (SDR) repeats, that is present in staphylococci [4]. In addition to ClfA and ClfB, *S. aureus* also expresses three SDR-proteins, SdrC, SdrD and SdrE, which are organized in tandem in the genome. These proteins are also thought to be involved in tissue colonization, and elimination of any of them decreases bacterial virulence [5]. Three additional members of this family, SrdF, SdrG and SdrH, are present in most *S. epidermidis* strains [6]. In each of these proteins, the SDR-region, which contains between 25 and 275 SD-dipeptide repeats, is located between the N-terminal ligand-binding A-domain and a C-terminal LPXTG-motif, which mediates anchoring to the cell wall by the transpeptidase sortase A. The function of the SDR-domain remains unknown, although it has been proposed to act as a cell wall spanning domain allowing exposure of the N terminal ligand binding sites of these proteins [7]. Serine rich glycoproteins have been identified in several other pathogenic bacteria, with demonstrated roles in bacterial adhesion. As yet, it remains unknown if *S. aureus* and *S. epidermidis* SDR-proteins are sugar modified and whether the SDR-domain contributes to virulence of staphylococci.

In the present study, we have discovered that SDR-domains of all SDR-proteins of *S. aureus* and *S. epidermidis* are heavily glycosylated by two novel glycosyltransferases, SdgA and SdgB. These glycosylation events prevent degradation of these proteins by host proteases, thereby preserving bacterial host tissue interactions. These sugar modifications also represent a dominant antibody epitope.

## Results

### Characterization of a highly opsonic monoclonal antibody (rF1) isolated from an MRSA infected donor

**Figure 1:**
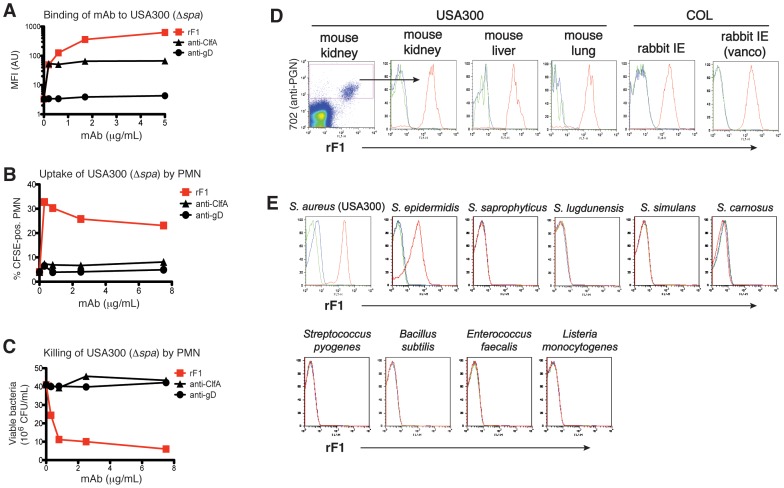
mAb rF1 exhibits robust binding to and killing of *S. aureus* bacteria. (**A-C**) Bacteria were preopsonized with huIgG1 mAbs rF1 (squares), 4675 anti-ClfA (triangles), or anti-herpes virus gD (circles). (**A**) Binding of mAbs to WT (USA300-Δ*spa*) bacteria was assessed by flow cytometry, and expressed as mean fluorescent intensity (MFI). (**B**) CFSE-labeled, preopsonized WT (USA300-Δ*spa*) bacteria were incubated with human PMN. Bacterial uptake was expressed as % of CFSE-positive PMN, after gating for CD11b-positive cells by flow cytometry. (**C**) Preopsonized WT (USA300-Δ*spa*) bacteria were incubated with PMN to assess bacterial killing. Numbers of viable CFU per mL are representative of at least three experiments. (**D**) Flow cytometry analysis of binding of rF1 to *S. aureus* from various infected tissues. Homogenized tissues were double stained with mAb rF1 (X-axis), and with anti-peptidoglycan mAb 702 to distinguish bacteria from tissue debris (Y-axis) (left panel; gate indicated by arrow), followed by gating of bacteria to generate histogram figures. (**E**) Binding of rF1 to various staphylococcal and non-staphylococcal Gram-positive bacterial species by flow cytometry. *Red lines*, rF1; *blue lines*, isotype control mAb anti-gD; *green lines*, control without mAb. (See also Figure S1).

We isolated several *S. aureus-*reactive monoclonal antibodies (mAb) from memory B cells from peripheral blood of MRSA-infected donors. When characterizing these antibodies, we identified one IgG1 mAb (hereafter referred to as rF1) with broad reactivity to a panel of *S. aureus* strains that induced robust opsonophagocytic killing (OPK) by human polymorphonuclear leukocytes (PMN). Maximum binding of mAb rF1 to bacteria from clinical MRSA strain USA300 was approximately 10 fold higher than that of an isotype-matched anti-ClfA mAb (Figure 1A). Consistent with increased binding, opsonization with rF1 resulted in increased uptake (Figure 1B) and killing (Figure 1C) of USA300 by PMN. In contrast, preopsonization with human anti-ClfA had no effect on bacterial viability (Figure 1C). The rF1 antibody did not affect viability of USA300 in the absence of PMN (not shown). Thus, rF1 is a mAb with the capacity to bind MRSA and induce potent killing of MRSA by PMN.

Flow cytometry (FCM) analysis showed potent binding activity of rF1 to all 15 *S. aureus* strains tested (Figure S1). These strains were broadly distributed across the *S. aureus* phylogeny [8]. As expression levels of bacterial cell surface antigens might differ between *in vitro* and *in vivo* growth, we also tested the ability of rF1 to recognize USA300 isolated from various mouse tissues after systemic infection. The rF1 mAb strongly bound to USA300 derived from infected mouse kidneys, livers and lungs (Figure 1D). The binding rF1 to USA300 from mouse kidneys was sustained until at least 8 days after infection (not shown), suggesting robust long-term expression of the rF1 epitope during infection. In addition, rF1 strongly bound to MRSA COL bacteria from heart vegetations in a rabbit model of infectious endocarditis. Treatment with vancomycin did not affect the reactivity of rF1 with MRSA (Figure 1D). Thus, the antigen recognized by rF1 is conserved across various strains and stably expressed in various growth and infection conditions.

Given the ubiquitous nature of rF1-reactivity across all *S. aureus* strains, we further queried if such reactivity is extended to other gram-positive bacteria. Notably, rF1 binding was detectable only for the coagulase-negative human pathogen *S. epidermidis* (Figure 1E). The rF1 mAb did not bind to any other staphylococcal species tested, including *S. saprophyticus*, *S. lugdunensis*, *S. simulans* and *S. carnosus*, or other Gram-positive species such as *Streptococcus pyogenes, Bacillus subtilis*, *Enterococcus faecalis*, and *Listeria monocytogenes* (Figure 1E). Thus, rF1 is a human antibody that binds to stably-expressed surface antigen(s) on human-adapted staphylococcal pathogens and promotes bacterial killing by human PMNs.

### mAb rF1 binds to the SDR region of staphylococcal SDR proteins

**Figure 2:**
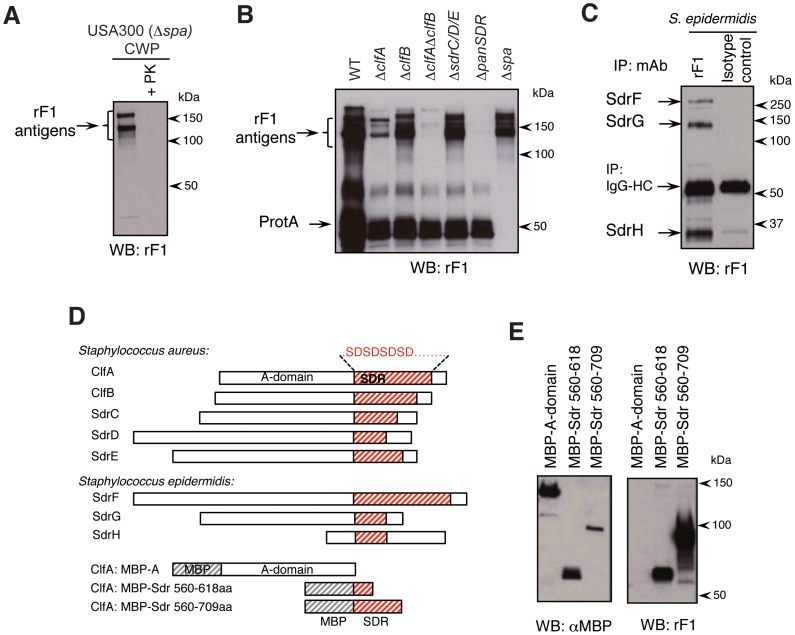
mAb rF1 binds to a family of serine-aspartate-repeat (SDR)-proteins. (**A**) rF1-reactivity with USA300 CWP is sensitive to proteinase-K (PK) treatment. Lysostapahin-derived CWP from WT (USA300-Δ*spa*) bacteria was left untreated (lane 1) or treated with 10 µg/mL PK for 1 hour (lane 2), and immunoblotted with rF1. (**B**) rF1-reacitivty is dependent on the presence of SDR-proteins. CWPs from WT, indicated deletion strains of various combinations of SDR-family proteins [12], [40], and a Δ*spa* strain as control for non-specific binding, were immunoblotted with rF1. The lower molecular weight bands (∼50 kDa) were due to non-specific IgG binding to protein A. (**C**) rF1 also binds to additional SDR-proteins from *S. epidermidis*. Cell lysates from *S. epidemidis* were immunoprecipitated with rF1 (lane 1) or an isotype-control mAb (lane 2) and immunoblotted with rF1 mAb. Identities of rF1-reactive bands were revealed by mass-spectrometry of the same lysates (see also Figure S2). (**D**) Alignment of SDR-proteins revealed by mass-spectrometry from *S. aureus* and *S. epidermidis*. SDR-regions are indicated by red hatches. Three truncation mutants of clumping factor A (ClfA) that were fused with maltose-binding protein (MBP) are also shown. (**E**) SDR-region is sufficient for rF1 reactivity. CWPs from *S. aureus* expressing truncated recombinant constructs were immunoblotted with anti-MBP mAb or rF1 mAb.

We next sought to identify the *S. aureus* antigen(s) responsible for rF1 reactivity. Immunoblotting of cell wall preparations (CWPs) with rF1 revealed that the mAb binds to a group of high molecular weight entities. Treatment of CWP with proteinase K completely eliminated rF1 reactivity, suggesting that the rF1 antigens are cell wall-associated proteins (Figure 2A). Furthermore, rF1 reactivity was not altered in CWPs derived from MRSA strains lacking some of the most abundant surface-expressed carbohydrate antigens, such as wall teichoic acids (Δ*tagO* mutant) and poly-N-acetyl glucosamine (Δ*icaA* mutant) (not shown).

As many as 21 proteins are attached to the cell wall by the sortase A enzyme [9]. Sortase A-anchored proteins include an interesting class of proteins that each contain a long tract of serine-aspartate (SD) repeats near the C-terminus; five of these proteins are present in *S. aureus* [10] and three in *S. epidermidis*. rF1 reactivity was abolished in a Δ*srtA S.aureus* strain [11] (not shown) and in a Δ*panSDR S.aureus* strain [12], in which all five SDR-proteins were deleted (Figure 2B). Binding of rF1 was significantly reduced in a Δ*clfA S.aureus* mutant, reflecting the abundance of cell wall-associated ClfA. We also probed the identity of rF1-reactive proteins in *S. epidermidis*, by immunoprecipitation of *S. epidermidis* lysates with rF1 followed by immunoblotting. Three unique rF1-reactive bands were present in *S. epidermidis* lysates, and mass spectrometry analysis of these bands identified peptides corresponding to SdrF, SdrG and SdrH (Figure 2C; Figure S2). These data suggest that the rF1 epitope is present on SDR proteins in both *S. aureus* and *S. epidermidis*.

To determine which specific region of SDR-proteins is recognized by rF1, we designed three different recombinant maltose binding protein (MBP)-tagged constructs based on the sequence of the *S. aureus* ClfA protein as a model antigen. These constructs contained either the N-terminal A-domain of ClfA, or the first 58 amino acids (SDR_560-618_) or the entire length of 150 amino acids (SDR_560-709_) of the SDR-domain of ClfA all under *tet*-inducible promoters (Figure 2D). Blotting with anti-MBP antibodies confirmed that each of these three recombinant proteins was expressed in *S. aureus* (Figure 2E, left panel). Only proteins containing SDR-domains were reactive with rF1 by immunoblotting, with increased binding in the strain expressing the longer stretch of SD-repeats (Figure 2E, right panel). Collectively, these observations indicate that rF1 epitopes reside on the SDR region of the SDR family of proteins of *S. aureus* and *S. epidermidis*.

### mAb rF1 binding requires species-specific post-translational modifications on SDR-domains

**Figure 3:**
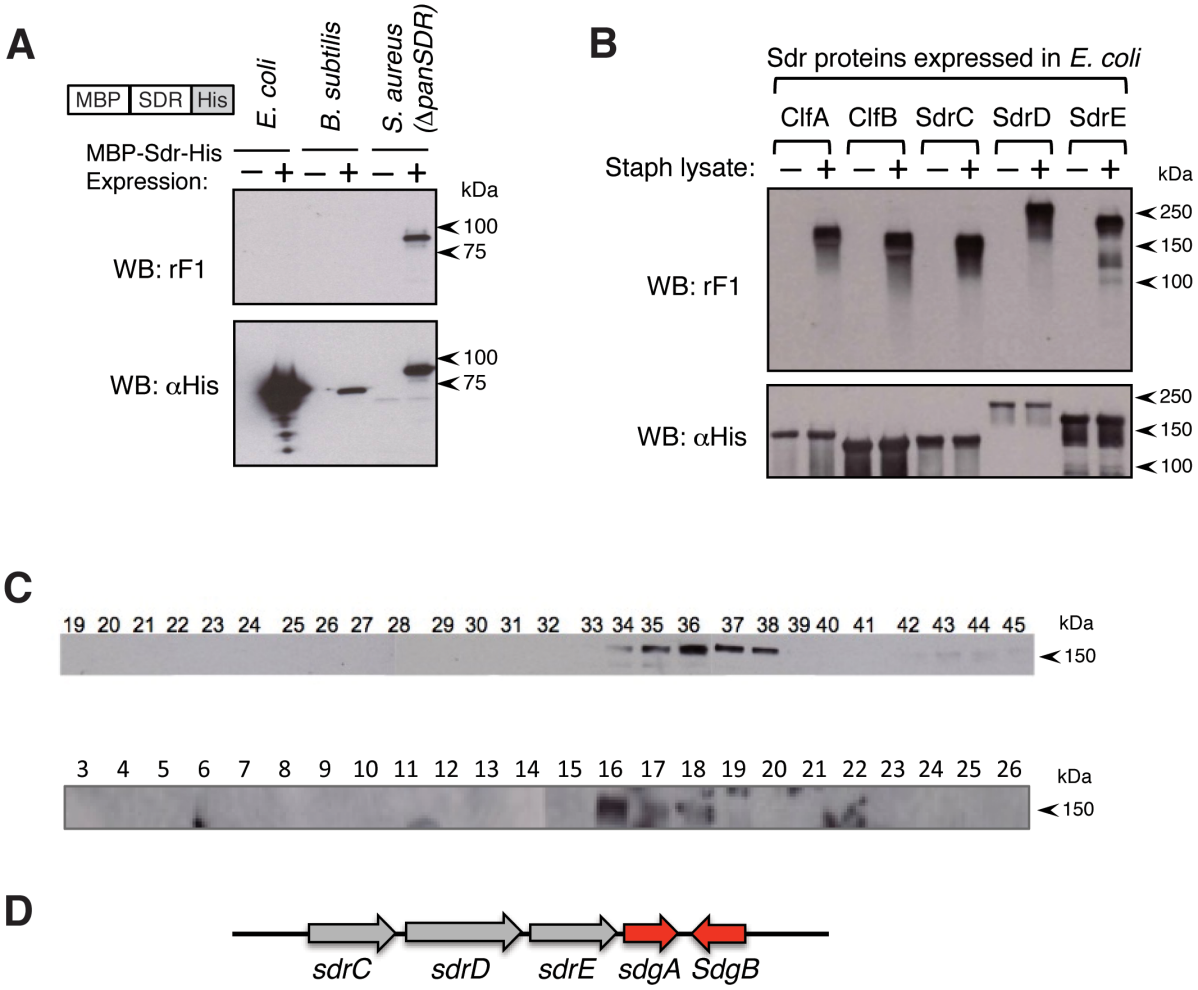
mAb rF1 recognizes post-translational sugar modifications on SDR-proteins. (**A**) rF1-reactivity is a pathogen-specific modification. His-tagged MBP-SDR constructs were over-expressed in *E. coli, B. subtilis*, or *S. aureus*, and whole cell lysates were immunoblotted with rF1 or anti-His mAb. (**B**) *E. coli* derived SDR-proteins can be post-translationally modified by *S. aureus* lysates to confer rF1-reactivity. His-tagged *E. coli*-expressed SDR-proteins were bound to nickel beads, incubated with or without Δ*panSDR* mutant *S. aureus* whole cell lysate and immunoblotted with rF1 or anti-His mAbs. (**C**) Fractionation of *S. aureus* cellular components conferring rF1-reactivity. His-tagged, *E. coli*-expressed ClfA protein was bound to nickel beads and incubated with fractions of Δ*panSDR* mutant *S. aureus* whole cell lysates that were prepared by size exclusion chromatography (upper panel), and further fractionated by ion-exchange chromatography (bottom panel). Individual fractions were spiked with *B. subtilis* lysate to provide necessary building blocks for the reactions. Beads were washed, eluted and immunoblotted with rF1. (See also Figure S3). (**D**) Genomic organization of the SDR-CDE locus. The glycosyltransferase genes *sdgA (SAUSA300_0549)* and *sdgB (SAUSA300_0550)* are found adjacent to the *sdr*-genes.

Since rF1 recognized *S. aureus* and *S. epidermidis*, but not other staphylococcal species or other genera of Gram positive organisms (Figure 1), we next analyzed rF1 reactivity with recombinant SDR_560-709_ domain of ClfA, containing N-terminal MBP and C-terminal His tag (MBP-SDR-His), which was expressed in Δ*panSDR S. aureus*, *B. subtilis* or *E. coli* to prevent reactivity of rF1 with endogenous SDR-family proteins. Immunoblot analysis using an anti-His antibody confirmed expression of MBP-SDR-His in all three bacterial species (Figure 3A, bottom panel). Surprisingly, we observed that rF1 reactivity with MBP-SDR-His could only be detected when the protein was expressed in *S. aureus*, but not when expressed in *E. coli* or *B. subtilis* (Figure 3A, upper panel). Notably, the recombinant protein also showed a size increase when expressed in *S. aureus* (Figure 3A, bottom panel). Given that the rF1-reactive recombinant SD_560-709_ proteins appeared larger than their predicted sizes, and that rF1 reactivity is specific to *S. aureus* and *S. epidermidis* (Figure 1), we hypothesized that rF1 reactivity with SDR-proteins requires species-specific post-translational modification.

Next, we investigated whether all five *S. aureus* SDR-proteins, ie. CflA, CflB, SdrC, SdrD and SdrE, are subjected to the same post-translational modification. Therefore, we expressed each of these five SDR-proteins (tagged with His) in *E. coli*, purified the proteins and incubated them with whole-cell lysates of Δ*panSDR S. aureus*. Expression of these five unmodified SDR-proteins in *E. coli* was confirmed using anti-His antibody (Figure 3B, bottom panel). Each of the five SDR-proteins, when expressed in *E. coli*, was devoid of rF1 reactivity. However, incubation of these proteins with *S. aureus* lysates completely restored rF1 reactivity (Figure 3B, top panel), supporting the requirement of *S. aureus*-specific post-translational modification to generate rF1 reactivity on SDR-proteins. Heat inactivation of the Δ*panSDR S. aureus* lysates abolished this effect (not shown), raising the possibility that SDR-protein modification is mediated by enzymatic activity.

### Fractionation of *S. aureus* lysates identifies putative glycosyltransferases creating rF1 epitopes on SDR proteins

To identify the putative enzyme factor(s) affording rF1 reactivity to the SDR-proteins, Δ*panSDR S. aureus* protein lysate was fractionated by size exclusion chromatography. The fractions were incubated with ClfA expressed in and purified from *E. coli*, and analyzed for the ability to induce rF1 reactivity by immunoblotting. *B. subtilis* lysate was added to this reaction to provide putative factor(s) necessary for efficient post-translational modifications, such as energy resources (ATP) and/or potential sugar building blocks, which were likely absent in the fractions of the Δ*panSDR S. aureus* lysates. As shown in Figure 3A, *B* *. subtilis* itself does not have the ability to generate rF1-reactive modifications, presumably due to the lack of the necessary enzyme(s). Using this *in vitro* reconstitution assay, we obtained strong enzymatic activity in fractions 35–38 (Figure 3C, upper panel). Fractions 35–38 were pooled and fractionated by ion-exchange chromatography to further enrich for the enzyme(s) capable of modifiying the SDR-proteins. Only fraction 16 showed significant enzymatic activity (Figure 3C, bottom panel), and this fraction was subjected to mass spectrometry analysis to identify the enzyme(s) responsible. Four glycosyltransferases (Gtfs) were enriched only within this fraction, three of which belong to the Group 1 family: TarM (SAUSA300_0939) and two novel Gtfs (SAUSA300_0549 and SAUSA300_0550); and one from the Group 2 family, TarS (SAUSA300_0252) [13] (Figure S3). These glycosyltransferases were not detected in the negative control mass spectrometry analysis of fraction 25.

TarM and TarS have been shown to function as glycosyltransferases appending N-acetylglucosamines (GlcNAcs) to the phospho-polyribitol backbone of wall teichoic acids in alpha- and beta- enantiomeric configurations, respectively [13], [14]. The other putative glycosyltransferases have not been characterized. Interestingly, the glycosyltransferases SAUSA300_0549 and SAUSA300_0550 are encoded by two divergently transcribed genes adjacent to the *sdrCDE* locus (Figure 3D) and this genetic arrangement is completely conserved across all sequenced *S. aureus* strains. Homologs of SAUSA300_0549 and/or SAUSA300_0550 are also present in *S. epidermidis* strains where they are also found near loci encoding for SDR-proteins. Based on their genomic locations and their potential roles in glycosylation of SDR-proteins (see below), we named *SAUSA300_0549* and *SAUSA300_0550*, *sdgA* and *sdgB*, respectively, for SD-repeat glycosyltransferases.

### SDR-family proteins are glycosylated by SdgA and SdgB

**Figure 4:**
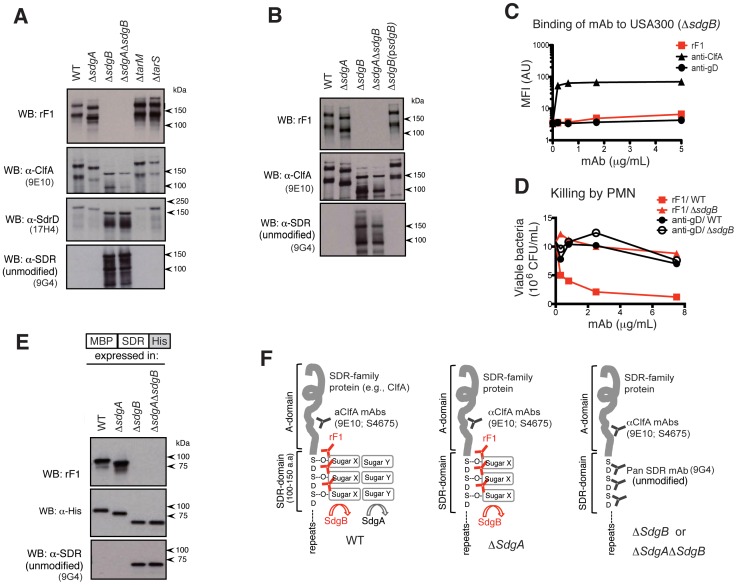
SdgB is the key rF1 epitope-modifying enzyme. (**A**) SdgB is necessary for rF1 reactivity. Cell wall lysates from WT and various putative glycosyltransferase mutants were immunoblotted with mAbs rF1, anti-ClfA (9E10), anti-SdrD (17H4) or anti-panSDR (9G4 α-SDR; recognizes the unmodified SDR-domain. (**B**) Complementation of Δ*sdgB* with exogenous SdgB confers rF1 reactivity. Cell wall lysates from WT, glycosyltransferase mutants, and the SdgB-complemented strain were immunoblotted with rF1, anti-ClfA, and anti-SDR mAbs as in (A). (**C**) Binding of rF1 to whole USA300 bacteria requires SdgB. Binding of mAbs to Δ*sdgB* USA300 was assessed by flow cytometry as described in Figure 1A. (**D**) rF1-mediated killing of USA300 activity requires SdgB. Wild-type USA300 bacteria preopsonized with rF1 (closed square) or anti-gD (closed circle), and Δ*sdgB* preopsonized with rF1 (closed triangle) or anti-gD (open circle), were incubated with PMN, and bacterial killing was determined as in Figure 1C. (**E**) MBP-SDR-His construct was expressed in WT, Δ*sdgA*, Δ*sdgB*, or Δ*sgdAΔsdgB S. aureus*, and whole cell lysates were immunoblotted with rF1, anti-His and anti-SDR. (**F**) Preliminary model for step-wise glycosylation of SDR-proteins by SdgB and SdgA. SDR-domains are first glycosylated by SdgB, which appends sugar modifications creating the epitope of mAb rF1. SdgA further modifies these epitopes with additional sugar moieties (left panel). The Δ*sdgA S. aureus* mutant shows that SdgA-mediated modifications do not influence rF1-binding activity (middle panel). In Δ*sdgB or* Δ*sgdAΔsdgB S. aureus*, the unmodified SDR-region is now recognized by the anti-pan-SDR mAb (9G4).

To verify which of these putative glycosyltransferases were responsible for modifying the SD-repeats, we generated *sdgA, sdgB*, *tarM*, or *tarS* deletion mutants in USA300, and assayed cell wall lysates from these mutants for reactivity with various antibodies including rF1. Strikingly, while all of these mutants remained proficient at expressing SDR-proteins, including ClfA and SdrD (Figure 4A, two middle panels), rF1 reactivity was completely lost in the absence of sdgB (Figure 4A, upper panel). Thus, rF1 reactivity with SDR-proteins requires SdgB. An antibody against unmodified SD-repeats reacted only with CWP from bacteria containing the Δ*sdgB* mutation, but not with WT and Δ*sdgA* bacteria (Figure 4A, bottom panel). This suggests that the SDR-domains are heavily decorated by SdgB-dependent sugar modifications, which disguise the protein backbone epitopes recognized by this antibody.

Complementation of Δ*sdgB* mutant with exogenous SdgB restored rF1 binding, and led to a concomitant loss of reactivity with the antibody against unmodified SD-repeats (Figure 4B). Overexpression of SdgB in the Δ*sdgB* mutant resulted in SDR-proteins migrating slower than those made by WT cells, presumably due to exaggerated sugar modification on these proteins by the transgene. Moreover, deletion of SdgB, abolished both binding of rF1 to whole bacteria, and rF1-induced bacterial killing by human PMN (Figure 4C and Figure 4D). Together, these data suggest that SdgB is the main protein that modifies SDR-proteins to enable recognition by rF1.

The apparent molecular weights of the SDR-proteins obtained from Δ*sdgA* were smaller compared to wild-type bacteria, and those from *ΔsdgB* were smaller than from Δ*sdgA* bacteria, as judged by immunoblotting (Figure 4A and 4B, top and middle panels). These differences likely reflect changes in relative molecular mass (M_r_) caused by sugar modifications. If the sugar modifications of SdgA and SdgB occured independent of each other, one expectation would be that SDR-proteins from Δ*sdgAΔsdgB* bacteria would run faster than from Δ*sdgB* bacteria. However, ClfA from Δ*sdgB* and *ΔsdgAΔsdgB* mutants showed a similar M_r_ (Figure 4A and 4B, middle panels), suggesting that the sugar modification appended by SdgA depends on SdgB activity.

Next, we tested the hypothesis that SdgA acts after SdgB, by immunoblotting whole cell lysates from wild-type, Δ*sdgA, ΔsdgB*, or *ΔsdgAΔsdgB* strain that expressed MBP-SDR-His under the control of a *tet*-inducible promoter. In both the Δ*sdgB* and *ΔsdgAΔsdgB* mutants, we observed reactivity with antibodies to unmodified SD-repeats (Figure 4E, bottom panel), but not with rF1 (Figure 4E, upper panel). The MBP-SDR-His proteins expressed in Δ*sdgB* and *ΔsdgAΔsdgB* mutants had a similar M_r_, as shown by both anti-SDR (Figure 4E, bottom panel) and anti-His antibodies (Figure 4E, middle panel). This supports the notion that no carbohydrate modification of the SDR-domains occurs in the absence of SdgB. In the Δ*sdgA* mutant, rF1 reactivity was unaffected; however, the SDR protein had a lower M_r_ than in wild-type cells. Together, these data suggest that SdgB and SdgA add sugar modifcations onto SDR-domains in a sequential manner, with SdgB appending the sugar residues proximal to the target Ser-Asp repeats, followed by additional modification by SdgA (see model, Figure 4F).

### SdgB and SdgA sequentially modify SDR-domains with GcNAc moieties

**Figure 5:**
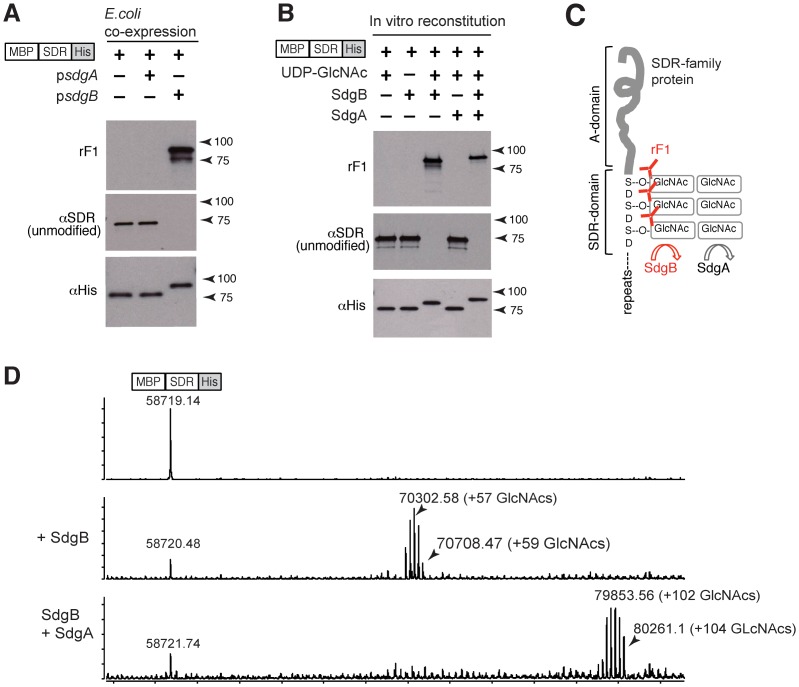
SdgB and SdgA sequentially modify the SDR-domain with GlcNAc moieties. (**A**) SdgB generates rF1 epitopes on SDR protein. A combination of MBP-SDR-His and SdgA or SdgB was co-expressed in *E. coli*, and cell lysates were immunoblotted with mAb rF1, or with mAb against unmodified SDR (9G4) or anti-His. (**B**) Cell-free system to reconstitute SDR glycosylation using purified components. Recombinant MBP-SDR-His was incubated with purified SdgA or SdgB, and in the presence or absence of UDP-GlcNAc; rF1 reactivity was induced only in the presence of SdgB and UDP-GlcNAc. (**C**) Final model for step-wise glycosylation of SDR proteins by SdgA and SdgB. First, SdgB appends GlcNAc moieties onto the SD-region on SDR proteins, followed by additional GlcNAc modification by SdgA. The epitope for mAb rF1 includes the SdgB-dependent GlcNAc moieties. (**D**) Mass spectrometry analysis to identify the SDR-sugar moieties using purified MBP-SDR-His expressed in *E. coli*. (Upper panel) Deconvoluted mass spectrum of purified MBP-SDR-His protein, showing the expected intact mass of 58719 Da. (Middle panel) MBP-SDR-His protein was treated with purified SdgB enzyme in the presence of UDP-GlcNAc for 2 h at 37°C. After incubation, the mass of the MBP-SDR-His protein showed several peaks, each peak being separated from the others by the mass of additional GlcNAc residues. (Bottom panel) The above-mentioned reaction mixture of MBP-SDR-His and SdgB (middle panel) was additionally treated with purified SdgA enzyme. After further incubation for 2 hrs at 37°C, up to an additional 47 GlcNAc groups were found to be added. Thus, most of the serines in the DSD motifs in MBP-SD can be modified with these disaccharide sugar moieties.

Next, we determined the nature of the sugar epitope that is added by SdgB and recognized by mAb rF1 using mass spectrometry. First, we co-expressed SdgA or SdgB together with MDP-SDR-His fusion protein as substrate in *E. coli*. Immunoblot analysis of the whole-cell lysates showed that rF1-reactivity on the MDP-SDR-His protein required expression of SdgB (Figure 5A, upper panel). The lack of reactivity of the *E. coli*-expressed protein with mAb against unmodified SDR proteins suggests that glycosylation by SdgB masks the backbone epitope for this antibody (Figure 5A, middle panel). Mass spectrometry of the purified MDP-SDR-His proteins from these *E. coli* bacteria suggested that SdgB was appending GlcNAc on these SDR-domains (data not shown).

When recombinant MBP-SDR-His fusion protein, purified from *E. coli*, was incubated with purified SdgA or SdgB, in the presence or absence of UDP-GlcNAc, the MBP-SDR-His substrate gained rF1-reactivity only in the presence of SdgB and UDP-GlcNAc (Fig. 5B). Alternative sugar resources such as UDP-Glucose and GDP-mannose did not confer rF1-reactivity (data not shown). As was shown in *E. coli*, SdgA alone had no effect on rF1 immunoreactivity or on the M_r_ of the recombinant substrate, but the combination of SdgB and SdgA resulted in more protein modifications than SdgB alone, as suggested by the higher M_r_ of SDR-substrate in rF1 and anti-His immunoblots (Figure 5B). Since this effect occurred when UDP-GlcNAc was the only sugar source, it is likely that SdgA as well as SdgB can append GlcNAc residues to SDR proteins, but SdgA does so only after SdgB modification has occurred (Figure 5C).

To determine the extent and complexity of SdgA and SdgB-mediated GlcNAc modification, enzyme-modified MDP-SDR substrates from the cell-free glycosylation reconstitution system were enriched by liquid phase chromatography and subjected to mass-spectrometric analysis (LC-MS). Compared to the unmodified MDP-SDR-recombinant substrate (Figure 5D, top panel), MDP-SDR-recombinant substrate that was modified by SdgB showed several peaks, with each peak separated from the others by mass of one additional GlcNAc residue (Figure 5D, middle panel). Here, the largest mass observed (70708 Da), represents the addition of 59 GlcNAc residues. In the MDP-SDR-recombinant substrate there are a total of 60 “Asp-Ser-Asp” (DSD) motifs. These data suggest that under these conditions, almost all the serines in the SDR-domain were modified with a GlcNAc residue. When the MDP-SDR-recombinant substrate was incubated with both SdgB and SdgA, up to an additional 47 GlcNAc residues were added (Figure 5D, bottom panel). These data suggest that most serines in the DSD-motifs are modified by disaccharide GlcNAc moieties through the sequential activity of SdgB and SdgA.

### SdgB-mediated glycosylation of SDR-proteins creates an immunodominant epitope for human antibodies

**Figure 6:**
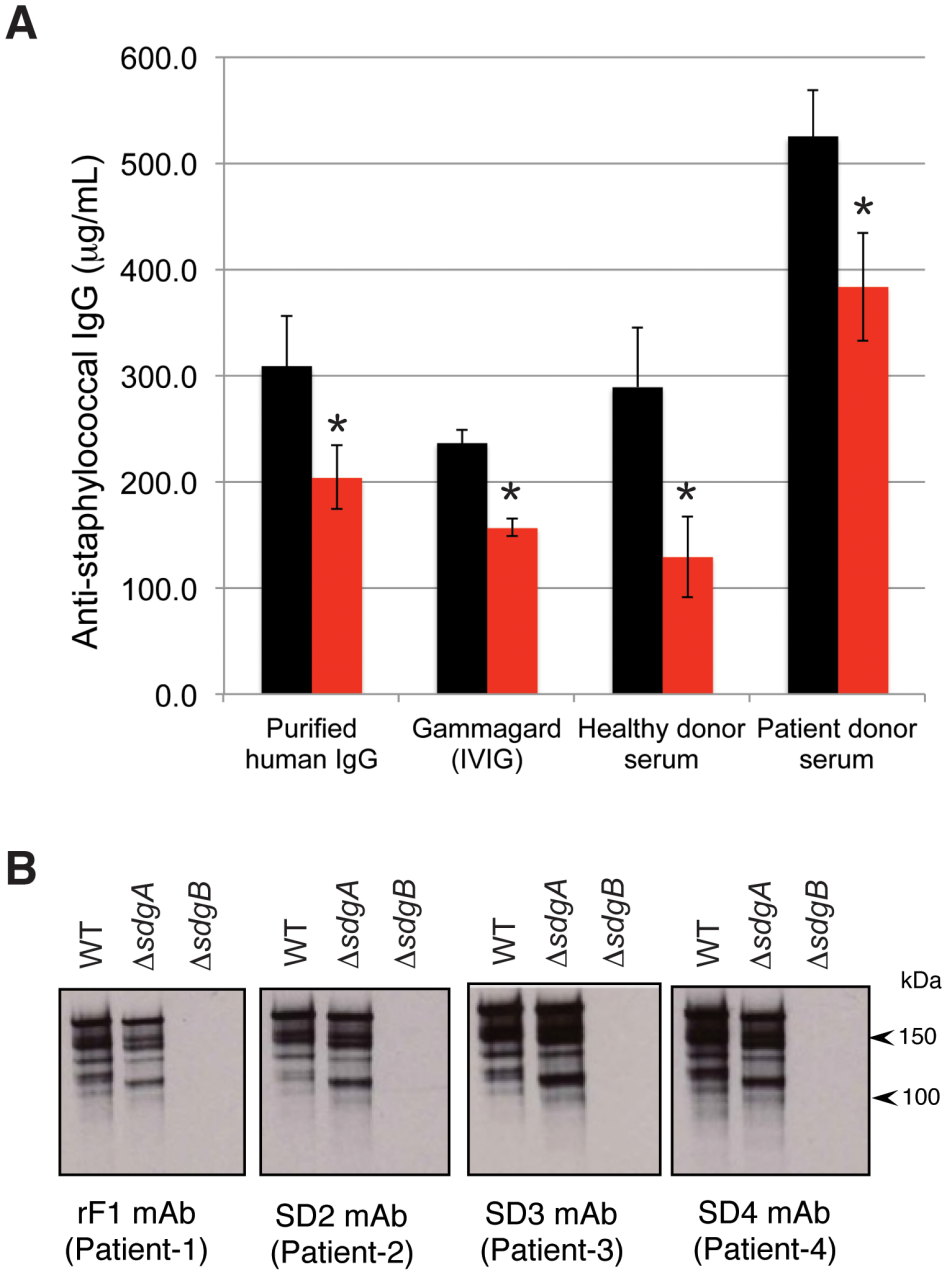
Recognition of SdgB-dependent epitope by human antibodies. (**A**) Four different human IgG preparations were reacted with plate-bound CWP from WT or Δ*sdgB* USA300 by ELISA. To calculate the specific anti-staphylococcal IgG content, data were normalized using a calibration curve with known IgG concentrations of a mAb against peptidoglycan, which has the same reactivity with both USA300 strains by ELISA. Data are expressed as µg/mL of anti-staphylococcal IgG in the serum. The reduction in reactivity observed for CWP from Δ*sdgB* (red bars) as compared to wild-type CWP (black bars) reflects IgG specific for SdgB-dependent epitopes. Asterisks indicate significant differences (p < 0.05) from WT CWP. (**B**) CWP from WT, Δ*sdgA*, or Δ*sdgB*, Δ*sdgAΔsdgB* USA300 were immunoblotted with rF1 and three additional human mAbs (SD2, SD3, and SD4) from different patients. All four mAbs showed similar epitope specificity.

Given our discovery of rF1 recognizing the SdgB-dependent glycosylation on SDR-proteins, we next determined whether this reflects a unique or rather common epitope specificity during anti-staphylococcal immune responses in humans. First, we analyzed the binding of human IgG from four different sources to cell wall preparations from either WT or Δ*sdgB* USA300 by ELISA. We tested (1) purified human IgG, (2) Gammagard, an intravenous immunoglobulin preparation purified from a plasma pool of ∼10,000 healthy donors [15], (3) serum pooled from healthy donors, and (4) serum pooled from MRSA patients. All four IgG preparations exhibited a significant reduction in reactivity with Δ*sdgB* CWP as compared to WT CWP (Figure 6A). Here, approximately 160 µg/mL of IgG in healthy serum and 140 µg/mL of IgG in patient serum accounted for the difference in reactivity with Δ*sdgB* compared to WT CWP. Given that the total IgG concentration in human serum fluctuates around 12 mg/mL, this indicates that the proportion of Sdg-dependent IgG can represent up to approximately 1% of total human serum IgG. This observation suggests that SdgB-dependent antibodies constitute a substantial proportion of the total anti-staphylococcal IgG content in humans, likely resulting from previous exposure to staphylococci.

Secondly, we were able to isolate three additional IgG mAb clones from three different *S. aureus* infected patients, which showed the same pattern of antigen recognition with wild-type or Δ*sdgA* USA300 CWP as mAb rF1, and which also showed absence of reactivity using Δ*sdgB* CWP (Figure 6B). Thus, we have found four independent IgG clones from four different patient donors, showing the same SdgB-dependent epitope specificity. Together, these data suggest that the SdgB-dependent glycosylation on SDR proteins reflects an immunodominant epitope for human anti-staphylococcal antibody responses.

### Glycosylation protects SDR proteins against proteolysis by human neutrophil-derived cathepsin G

**Figure 7:**
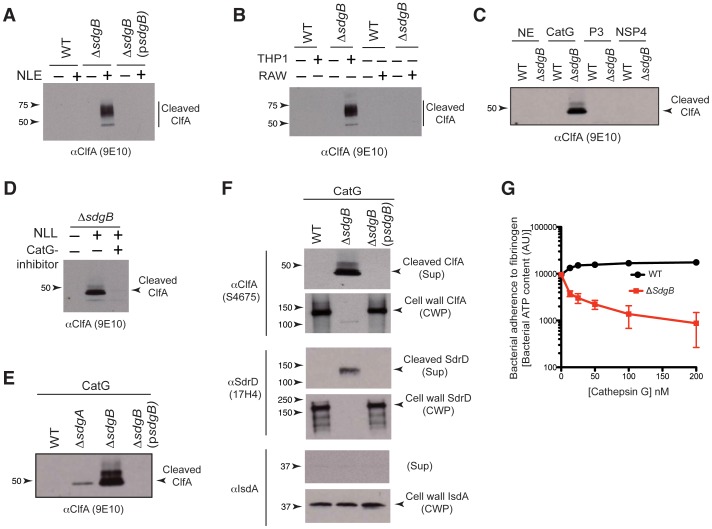
SdgB glycosylation protects SDR proteins from cleavage by human neutrophil-derived cathepsin G. (**A**) Live, in tact WT or Δ*sdgB* USA300 bacteria were incubated in the presence or absence of human neutrophil lysosomal extracts (NLE). Culture supernatants were immunoblotted with a mAb against the A-domain of ClfA (9E10) to detect cleaved ClfA fragments released from the bacteria. (**B**) Live, in tact WT or Δ*sdgB* cells were incubated in the presence or absence of lysosomal extracts from human THP1 cells or mouse RAW cells and culture supernatants were immunoblotted with anti-ClfA. (**C**) Live, intact WT or Δ*sdgB* cells were incubated with a panel of purified human neutrophil serine proteases, ie. neutrophil elastase (NE), cathepsin G (CatG), proteinase-3 (P3), and neutrophil serine protease-4 (NSP4). (**D**) **Δ** *sdgB* cells were treated with human neutrophil lysosomal extract in the presence or absence of a biochemical inhibitor of cathepsin G. (**E**) WT or various Sdg-mutant strains were treated with purified human cathepsin G. (**B-E**) Culture supernatants were analyzed by immunoblotting as in (A) to detect released ClfA fragments. (**F**) Live bacteria of WT, **Δ** *sdgB*, or Δ*sdgB* complemented with exogenous SdgB (p*sdgB*) were treated with purified human cathepsin G. Culture supernatants (Sup) or cell wall preparations (CWP) were immunoblotted with mAb against the A-domain of ClfA (S4675), SdrD (17H4), or IsdA (2D3). In addition to S4675, another mAb against the A-domain of ClfA (9E10) showed similar results (not shown). (**G**) Human cathepsin G inhibits adherence of glycosylation-deficient *S. aureus* to human fibrinogen. Live WT or Δ*sdgB* USA300 bacteria were pre-incubated with cathepsin G, and allowed to adhere to fibrinogen-precoated plates. Bacterial adhesion was quantified by measuring the amount of bacterial ATP associated with the plates.

For successful colonization and invasion of host tissues, *S. aureus* bacteria must evade attack by a variety of host immune mechanisms, including proteolytic enzymes derived from neutrophils. In this context, we analyzed the physiological roles of the GlcNAc modification of SDR proteins during interaction of the bacteria with neutrophil lysosomal enzymes. A large cleaved fragment of ClfA was released when intact Δ*sdgB* bacteria was incubated with a human neutrophil lysosomal extract, but this fragment was not cleaved from WT USA300 or from Δ*sdgB* bacteria complemented with exogenous SdgB (Figure 7A). Similar results were observed using lysosomal extracts derived from the human monocytic cell line THP1, but not from mouse monocytic cell line RAW (Figure 7B). Mass spectrometry analysis revealed that lysosomal extracts from both human neutrophils and human THP-1 cells were highly enriched for cathepsin G. In contrast, mouse RAW cells showed abundant expression of other cathepsin family members, but not cathepsin G (not shown). Thus, these data suggested that SdgB-mediated glycosylation protects ClfA against proteolytic activity of the human neutrophil lysosomal enzyme cathepsin G.

To determine which protease(s) is responsible for cleaving unglycosylated ClfA, we incubated WT or Δ*sdgB* cells with each of the four main human neutrophil-derived serine proteases, ie. neutrophil elastase, cathepsin G, proteinase-3 and neutrophil serine protease-4. Of these four proteases, only cathepsin G was able to induce cleavage of unglycosylated ClfA from Δ*sdgB* cells (Figure 7C). In addition, none of the serum serine proteases α-thrombin, plasmin, kallikrein, factor Xa, or factor X1a, were able to induce cleavage of unglycosylated ClfA (not shown). A biochemical inhibitor of cathepsin G abrogated cleavage of unglycosylated ClfA by neutrophil lysosomal extracts (Figure 7D), confirming that cathepsin G is the predominant enzyme mediating this effect. *S. aureus* expressing SdgB but lacking SdgA treated with cathepsin G showed less ClfA cleavage than Δ*sdgB* bacteria (Figure 7E), indicating that GlcNAc disaccharides may provide more protection from proteolysis than GlcNAc monosaccharide modifications.

Next, we analyzed the extent of reduction in the amount of cell surface-associated ClfA resulting from cathepsin G-mediated cleavage in the absence of glycosylation. The release of cleaved ClfA into the supernatant of Δ*sdgB* cells correlated with an almost complete loss of cell-associated ClfA (Figure 7F, upper two panels). We also observed that this effect is not limited to ClfA, since SdrD, another member of the SDR family, was similarly sensitive to cathepsin G-induced cleavage and release from Δ*sdgB*, but not WT *S. aureus* cells (Figure 7F, middle two panels). However, this cathepsin G-mediated cleavage appeared restricted to the Sdr-family proteins, since IsdA, another sortase-A anchored cell surface protein, was not affected (Figure 7F, bottom two panels). Together, these data demonstrate that cell surface-bound SDR proteins are sensitive to proteolysis by human cathepsin G, and that glycosyl modification, primarily by SdgB, protects against this activity.

Finally, we hypothesized that the observed cathepsin G-mediated disappearance of unglycosylated SDR proteins from the bacterial surface would have biological consequences, such as the capacity of the bacteria to adhere to host tissues. To test this hypothesis, we analyzed the effects of cathepsin G on the adherence of SDR glycosylation-competetent or -deficient bacteria to human fibrinogen, a well-known host ligand for SDR proteins including ClfA [2]. Treatment with cathepsin G inhibited the adherence of Δ*sdgB*, but not WT bacteria to human fibrinogen (Figure 7G). Thus, SdgB-mediated glycosylation of SDR proteins likely represents a bacterial mechanism of protection against host innate immune responses to safeguard successful tissue colonization.

## Discussion

The data presented in this study highlight three novel findings: (1) the identification and characterization of two novel glycosyltransferases, designated SdgA and SdgB, which glycosylate serines in the Ser-Asp motifs of all SDR-proteins in *S. aureus* by appending GlcNAc moieties; (2) the elucidation of a protective role for these glycosylation events against proteolysis of SDR-proteins by human neutrophil cathepsin G; and (3) the discovery of highly opsonic human antibodies that are directed against these GlcNAc-modifications.

The glycosyltransferases SdgA and SdgB identified here function sequentially to append GlcNAc moieties onto SDR-proteins. Based on reactivity of the patient-derived, glycosylation-specific monoclonal antibody rF1 with Δ*sdgA*, Δ*sdgB* and Δ*sdgAΔsdgB* mutant bacteria, we propose a step-wise model, in which SdgB first appends GlcNAc on serine residues within the SD-repeats of SDR (Figure 5C). The first SdgB-dependent GlcNAc modification forms the minimal epitope recognized by rF1. This was confirmed in a cell-free glycosylation reconsititution assay using UDP-GlcNAc as the sole sugar donor; the nature of the modified substrate was confirmed by LC-MS analysis. The SdgB-dependent modification is followed by the addition of GlcNAc to the glycoproteins by the second enzyme, SdgA, yielding disaccharide GlcNAc moieties on SDR proteins. This is consistent with our observation that SDR-proteins bind succinylated wheat germ agglutinin (sWGA) lectin, which is known to require divalent GlcNAcs [16], only when both SdgB and SdgA are expressed (data not shown). Among all the identified glycosyltransferases (Gtfs), SdgB and SdgA are the most homologous to each other (43% identity; 63% similarity). TarM is another glycosyltransferase that appends GlcNAcs to wall teichoic acids; interestingly, TarM is the second most homologous protein to both SdgB (23% identity; 46% similarity) and SdgA (24% identity; 46% similarity).

In *S. aureus*, the genes for SdgA and SdgB are located adjacent to the SdrC/D/E locus. Some *S. aureus* strains, which lack some of the individual Sdr genes (SdrC or SdrD) or all three of them, still contain SdgA and SdgB. In addition to appending GlcNAcs to SdrC, D and E proteins, SdgA and SdgB append similar modifications to ClfA and ClfB, and these two genes are independent of each other and away from the SdrC/D/E locus. *sdgA* and *sdgB* genes are intact in all sequenced *S. aureus*; all 15 *S. aureus* and *S. epidermidis* strains that we have analyzed express rF1 antigen, suggesting that these genes are expressed and that this is a highly conserved property of these bacteria. Interestingly, as in *S. aureus*, the *sdgA* and *sdgB* homologs in *S. epidermidis* are usually adjacent to genes encoding SDR-proteins. The Sdg orthologue that is encoded adjacent to *S. epidermidis* SdrG, is most homologous to *S. aureus* SdgB (56% identity), which further explains why rF1 antibody reacts to modifications that are tailored by very similar Gtfs in both species. In some *S. epidermidis* strains, a *sgdA* homolog appears to be absent, suggesting that the second glycosylation step may be less conserved in *S. epidermidis*, perhaps consistent with its decreased invasiveness and consequent decreased need to evade neutrophil proteases. rF1 reactivity is only found in *S. aureus* and *S. epidermidis*, but not other staphylococci or gram-positive or -negative strains, and homologs of SdgA and SdgB are absent from all staphylococcal strains that were negative for rF1 binding. These lines of evidence support the notion that the sugar modifications of SDR-proteins may be specific for human-adapted staphylococci.

Cells of the innate immune system such as neutrophils and macrophages play a vital role in immediate defense against invasive staphlylococal infections. For successful establishment of mucosal colonization and for deeper infection *S. aureus* must be able to survive attack by a variety of potent antimicrobial substances, including reactive oxygen and nitrogen species, lysozyme, defensins and cathepsins. While *S. aureus* has mechanisms for evasion of each of these phagocyte host defenses, the work in this paper describes a novel one, since glycosylation of SDR proteins is also important in this regard. The glycosylation mediated by SdgA and SdgB protects SDR-proteins from proteolytic cleavage by cathepsin G produced by neutrophils and macrophages. SDR-proteins belong to a family of bacterial proteins collectively reffered to as MSCRAMMs (microbial surface components recognizing adhesive matrix molecules) which mediate bacterial adherence to host extracellular matrix components including fibrinogen, fibrinectin and collagen. Cleavage of SDR-proteins from the bacterial cell surface in this environment is likely to compromise successful establishment of *S. aureus* colonization of and proliferation in its exclusively human niche.

Glycosylation of adhesins of gram positive and gram negative pathogens is an emerging theme [17]. Glycosylation is necessary for the maturation and function of the SraP-like glycoproteins of gram-positive pathogens, and of the *E. coli* adhesins TibA and adhesin involved in diffuse adherence (AIDA) [17], [18], [19]. Similar to *S. aureus* SdgB and SdgA, the *E. coli* glycosyltransferase genes are found directly adjacent to the genes encoding their specific target proteins. The *S. aureus* SDR-proteins are distinct from several other glycosylated serine-rich adhesins of gram-positive bacteria, such as fimbriae-associated protein Fap1 of *Streptococcus parasanguinis* [20], SrpA of *Strep. sanguinis* [21], GspB of *Strep. gordonii* [22] and SraP of *S. aureus* [23]. Unlike these serine-rich proteins, the SDR-proteins have only one single serine repeat region, almost always with aspartate as the alternate amino acid, and they do not cluster with the accessory Sec apparatus. SgdA and SgdB do not have signal sequences or transmembrane domains, suggesting that they function in the cytoplasm, prior to the export of glycosylated SDR-proteins to the cell surface by an as yet unknown mechanism. In some of these bacterial species, glycosylation of serine-rich repeats, for example in *S. parasanguinis* Fap1 protein [24] or in the pneumococcal protein PsrP [25], has been associated with biofilm formation. In *S. aureus*, ClfB has recently been implicated in biofilm formation as well [26]. However, we have not observed attenuation in *in vitro* biofilm formation by the Δ*sdgAΔsdgB S. aureus* strain (not shown), which may be explained by functional redundancy with other glycosyl transferases appending similar sugar moieties on other cell wall associated molecules. Examples of this include TarM and TarS that append similar GlcNAcs on WTA molecules. However, glycolsyation events may also protect other bacterial functions. For instance, SdrE has been shown to bind complement factor H as an immune evasion mechanism [27], which may well be preserved by glycosylation-mediated protection of SdrE.

The precise roles of SDR glycosylation during infection remain to be defined. Since cathepsin G-mediated degradation of unglycolsylated SDR proteins results in the release of a proteolytic fragment containing the adhesive A-domain of SDR-proteins, we proposed that this cleavage could compromise the capacity of *S. aureus* to colonize host tissues. Supporting this hypothesis, we demonstrated that in the absence of glycosylation of SDR-proteins, human cathepsin G inhibits bacterial adhesion to human fibrinogen. Thus, we propose that Sdg-mediated glycosylation of SDR proteins is a bacterial mechanism of protection against host innate immunity, to safeguard efficient host tissue colonization. In a systemic mouse infection model, we have observed a minor, though non-significant, reduction in bacterial burden in kidneys for USA300 deletion strains lacking SdgA and SdgB (not shown). However, the significance of these glycosyl modifications may be underappreciated in mouse models. We showed that unglycosylated SDR-proteins in SdgA- or SdgB-deficient bacteria are susceptible to degradation by purified human cathepsin G, or by lysosomal extracts from human neutrophils or from a human monocytic cell line, but not from a mouse monocytic cell line. Thus, the lack of a strong phenotype of SdgA/B deficient *S. aureus* bacteria in mouse infection may be explained by the possibility that mouse cathepsin G is either less effective in degrading unglycosylated SDR proteins or present at lower levels than in human phagocytes. This is in agreement with previous studies showing divergence of human and mouse cathepsin G with respect to their substrate specificities [28], and normal infection of *S. aureus* in cathepsin G-deficient mice [29].

Since *S. aureus* and *S. epidermidis* are typical human pathogens, it is thus conceivable that the functions of these glycosylation events are important for infection in humans but not in mice. This is reminiscent of recent studies showing essential *S. aureus* virulence mechanisms, mediating specific interactions between *S. aureus* and host factors, to be specific for human and not occur in mice [30], [31]. A definite analysis of our hypothesis of a role for glycosylation in tissue colonization is technically difficult, due to the lack of appropriate *in vitro* models for *S. aureus* colonization of relevant human tissues, such as nasal or skin epithelium. We have observed that the purified bacterial protease Asp-N, a metallo-endoprotease from either *Pseudomonas fragi* or *Flavobacterium menigosepticum*, that hydrolizes the N-terminal side of aspartic acids shows enhanced cleavage of A-domains of SDR proteins from Δ*sdgB* mutants, compared to the wildtype S. aureus (data not shown). In this example, the track of “naked” Ser-Asp repeat residues on Δ*sdgB* mutants is a clear target for these proteases. This suggests that, in addition to interfering with host proteases, the glycosylation may protect S. aureus from proteases secreted by other bacteria competing for the same ecologic niche.

We demonstrated that the SdgB-mediated GlcNAc modification generates an epitope on SDR proteins that is specifically recognized by a patient-derived mAb rF1. This antibody induced robust opsonophagocytic killing by human neutrophils, much more potent than another human mAb that recognizes the A-domain of ClfA. The superior capacity of rF1 to induce killing can likely be explained by a higher abundance of glycosyl groups on the SDR-proteins. We also found that the SdgB-dependent GlcNAc-containing epitope on SDR-proteins is recognized by a significant proportion of the total amount of anti-staphylococcal IgG in humans, and that multiple patients are able to recognize this epitope. Thus, the SdgB-dependent GlcNAc modification of SDR-proteins represents an immunodominant epitope during human anti-staphylococcal immune responses. The SDR-glycosylation may be used by the host immune system as an important recognition factor to detect *S. aureus* in infected tissues. However, the potential importance of glycosylation of SDR proteins in protecting against host proteases and successful tissue colonization may outweigh the need of the bacteria to go undetected from the host antibody response, thus representing a balance between host and pathogen.

## Materials and Methods

### Ethics statement

Informed written consent was obtained from all donors and was provided in accordance with the Declaration of Helsinki. Approval was obtained from the health research ethics committee of Denmark through the regional committee for The Capital Region of Denmark, and the medical ethical committee of Academic Medical Center, Amsterdam.

All animal procedures were conducted under a protocol (#08-1990) approved by the Genentech's Institutional Animal Care and Use Committee in an AAALAC-accredited facility in accordance with the Guide for the Care and Use of Laboratory Animals and applicable laws and regulations.

### Bacterial strains and culture

Bacterial strains are listed in Table 1. For generation of mutant strains, we used protein A deficient (Δ*spa*) USA300 as parental strain, being referred to as wild-type USA300, in order to minimize non-specific antibody binding. Single *SgdA* and *SdgB* mutants were generated by transduction using phage Φ85 (ATCC, Manassas, VA) of transposon-inserted *SdgA* NE381 and *SdgB* NE105 genes from Nebraska Transposon Library strains (NE381 and NE105, respectively; obtained from NARSA) into Δ*mcr* USA300 NRS384 [32] or Δ*spa* USA300 NRS384. The *SdgAB* double mutant was generated by allelic exchange using the pIMAY plasmid (a generous gift from Dr. T. Foster) as previously described [32]. Briefly, a targeting construct including ∼600 bp of the upstream and downstream flanking sequences of the two adjacent *SdgA* and *SdgB* genes was cloned into pIMAY, which was electroporated into Δ*mcr* USA300 NRS384 or Δ*mcr*Δ*spa* USA300 NRS384.

**Table 1:** Strains, plasmids, and antibodies.

Strain	Genotype and/or description	Source or reference
NRS384	USA300 wild-type (MRSA clinical isolate)	NARSA*
	Newman (MSSA clinical isolate)	Dr. T. Foster
NRS100	COL (MRSA clinical isolate)	NARSA
NRS123	USA400 (MRSA clinical isolate)	NARSA
NRS382	USA100 (MRSA clinical isolate)	NARSA
NRS483	USA1000 (MRSA clinical isolate)	NARSA
NRS71	MRSA252 (MRSA clinical isolate)	NARSA
NRS70	N315 (MRSA clinical isolate)	NARSA
NRS102	Reynolds (MSSA clinical isolate)	NARSA
NRS103	Becker (MSSA clinical isolate)	NARSA
NRS148	Smith Diffuse (MSSA clinical isolate)	NARSA
NRS112	MN8 (MSSA clinical isolate)	NARSA
NRS1	Mu50 (VISA clinical isolate)	NARSA
29213	Rosenbach (MSSA clinical isolate)	ATCC
85W1740	*Staphylococcus epidermidis*	Ward's Natural Science
29970	*Staphylococcus haemolyticus*	ATCC
43809	*Staphylococcus lugdunensis*	ATCC
27848	*Staphylococcus simulans*	ATCC
51365	*Staphylococcus carnosus*	ATCC
85W1640	*Bacillus subtilis*	Ward's Natural Science
85W1743	*Enterococcus faecalis*	Ward's Natural Science
85W1746	*Streptococcus pyogenes*	Ward's Natural Science
43251	*Listeria monocytogenes*	ATTC
BL21(DE3) RIL	*Escherichia coli*	New England Biolabs
SKM1	Newman	(12)
SKM3	SKM1 Δ*srtA*:*ermC*	(12)
DU5999	Newman *clfA5* ClfA-	(14)
DU5962	Newman *clfB*:*LacZ* ClfB-	(30)
DU6000	Newman *clfA5 clfB*:*LacZ*[Ermr]	(14)
	Newman Δ*sdrCDE*:Tcr	(14)
DU6001	Newman *clfA5 clfB*:*LacZ*[Ermr] Δ*sdrCDE*:TcR	(14)
SM95	NRS384 *Δmcr*	(20)
SM41	NRS384 Δ*spa*	This study
SM69	NRS384 Δ*spaΔsdgA*	This study
SM54	NRS384 *ΔspaΔsdgB*	This study
SM195	NRS384 Δ*mcrΔspaΔsdgAΔsdgB*	This study
SM151	NRS384 *ΔmcrΔspaΔtarM*	This study
SM64	NRS384 Δ*spaΔtarS*	This study
SM122	NRS384 Δ*mcrΔsdgAΔsdgB*	This study
SM167	NRS384 *ΔmcrΔspaΔsdgB (*p*sdgB*)	This study
**Construct**		
KK812	pTet.MBP*-ClfA* -A-domain (Inducible expression in *S. aureus* of MBP fused to A-domain of ClfA (S40-G538); C-term 6x His-Tag; pTet vector)	This study
KK813	pTet.MBP-*ClfA-*Sdr560-618 (Inducible expression in *S. aureus* of MBP fused to the ClfA sdr region (D560-A618); C-term 6x His; pTet vector)	This study
KK814	pTet.MBP-*ClfA-*Sdr560-709 (Inducible expression in *S. aureus* of MBP fused to the ClfA sdr region (D560-S709); C-term 6x His: pTet vector	This study
KK774	pTet.MK4.MBP-*ClfA-*Sdr560-709 (Inducible expression in *S.aureus* and B. subtilis of MBP fused to the ClfA sdr region (D560-S709); C-term 6x His; pTet.MK4)	This study
KK699	pMal.c5x.MBP-*ClfA-*Sdr560-709 (Inducible expression in *E. coli* of MBP fused to the ClfA sdr region (D560-S709); C-term 6x His; pMal.c5x)	This study
KK815	ST239.*ClfA* (Inducible expression of N-term Unizyme Tag fused to the predicted mature start of the ClfA; ST239 *E. coli* vector)	This study
KK816	ST239.*ClfB* (Inducible expression of N-term Unizyme Tag fused to the predicted mature start of the ClfB; ST239 *E. coli* vector)	This study
KK817	ST239.SdrC (Inducible expression of N-term Unizyme Tag fused to the mature start of the SdrC; ST239 *E. coli* vector)	This study
KK818	ST239.SdrD (Inducible expression of N-term Unizyme Tag fused to the mature start of the SdrD; ST239 *E.coli* vector)	This study
KK819	ST239.SdrE (Inducible expression of N-term Unizyme Tag fused to the mature start of the SdrE; ST239 *E. coli* vector)	This study
KK820	pET.SdgA (Inducible expression of N-term 6x His Tag fused to the full length SdgA protein; pET15b *E. coli* vector)	This study
KK821	pET.SdgB (Inducible expression of N-term 6x His Tag fused to the full length SdgB protein; pET15b *E. coli* vector)	This study
KK822	pET.TarM (Inducible expression of N-term 6x His Tag fused to the full length TarM protein; pET15b *E. coli* vector)	This study
KK823	pET.TarS (Inducible expression of N-term 6x His Tag fused to the full length TarS protein; pET15b *E. coli* vector)	This study
KK803	pSarA.MK4 NT-His SdgB (Constitutive expression of N-term 6x His Tag fused to full length SdgB; pSarA.MK4 *S. aureus* complementation vector)	This study
KK698	pMal.c5x.MBP-*ClfA-*Sdr560–844 (Inducible expression in *E. coli* of MBP fused to the ClfA sdr region (D560-G844); C-term 6x His; pMal.c5x)	This study
KK824	pMal.c5x.MBP *-ClfA* -A-domain (Inducible expression in *S. aureus* of MBP fused to A-domain of ClfA (S40-G538); C-term 6x His-Tag; pMal.c5x)	This study
KK826	pMal.c5x.MBP *-ClfA* -A-domain (Inducible expression in *S. aureus* of MBP fused to A-domain of ClfA (S40-G300); C-term 6x His-Tag; pMal.c5x)	This study
KK828	pMal.c5x.MBP *-ClfA* -A-domain (Inducible expression in *S. aureus* of MBP fused to A-domain of ClfA (S40-S197); C-term 6x His-Tag; pMal.c5x)	This study
KKE852	pBad33.SdgB-(Inducible expression in *E. coli* of N-term 6x His Tag fused to the full length SdgB protein; pBAD33 *E. coli* Vector)	This study
KKE865	pBad33.SdgA-(Inducible expression in *E. coli* of N-term 6x His Tag fused to the full length SdgA protein; pBAD33 *E. coli* Vector)	This study
**Antibodies**		
rF1	human IgG1 mAb anti glycosylated SDR proteins	This study
SD2	human IgG1 mAb anti glycosylated SDR proteins	This study
SD3	human IgG1 mAb anti glycosylated SDR proteins	This study
SD4	human IgG1 mAb anti glycosylated SDR proteins	This study
gD:5237	human IgG1 mAb anti HSV gD protein	Genentech
4675	human IgG1 mAb anti ClfA	This study
9E10	mouse IgG1 mAb anti ClfA	This study
17H4	mouse IgG2b mAb anti SdrD	This study
2D3	mouse IgG2b mAb anti IsdA	This study
9G4	mouse IgG1 mAb anti unmodified SDR proteins	This study
28.9.9	rabbit IgG mAb anti PGN	This study
631212	mouse IgG2a mAb anti His	Clontech
E8038S	mouse IgG2a mAb anti MBP-HRP	New England Biolabs

Abbreviations: MRSA, methicillin-resistant *Staphylococcus aureus*; MSSA, methicillin-sensitive *Staphylococcus aureus*; VISA, vancomycin intermediate-resistant *Staphylococcus aureus*; mAb, monoclonal antibody. *NARSA, network on Antibiotic Resistance in *Staphylococcus aureus* (NARSA) Program supported under NIAID/NIH Contract No. HHSN272200700055C.

To complement the *SdgB* gene inΔ*spa*Δ*SdgB* USA300 NRS384, a construct containing the ribosomal binding site of the *SodA* gene followed by a His-tag fused in frame with the full length *SdgB* gene was cloned downstream of a constitutive *SarA* promoter in the p*SarA*.MK4 plasmid (ATCC). This plasmid was electroporated into Δ*spa*Δ*SdgB* USA300 NRS384, and complementation of the *SdgB* gene was confirmed by PCR.

Bacteria were grown on tryptic soy agar plates supplemented with 5% sheep blood (TSA plates) for 18 h at 37°C. For liquid cultures, single colonies from TSA plates were inoculated into tryptic soy broth (TSB) and incubated at 37°C while shaking at 200 rpm for 18 h; 100 fold dilutions of these cultures in fresh TSB were further subcultured for various times.

### Generation of antibodies

For generation of mAb rF1, CD19^+^CD3^−^CD27^+^IgD^−^IgA^−^ memory B cells were isolated from peripheral blood of an MRSA-infected donor using a FACSAria cell sorter (BD, San Jose, CA). Before viral transduction with B-cell lymphoma (Bcl)-xL and Bcl-6 genes, the memory cells were activated on CD40L-expressing mouse L fibroblasts in the presence of interleukin-21, as described previously [33]. Transduced B cells were maintained in the same culture system. The use of donor blood was approved by the institutional committee. rF1 was selected from culture supernatants by reactivity with lysates of MSSA strain Newman by ELISA; positive wells were subcloned and re-tested by ELISA twice. Recombinant rF1 was generated by cloning the heavy and light chain variable regions with human IgG1 kappa constant regions using pcDNA3.1 (Invitrogen) and transfection into 293T cells (ATCC). Purified IgG was obtained from culture supernatants using protein A-coupled sepharose (Invitrogen).

The human IgG antibodies 4675, SD2, SD3 and SD4 were cloned from peripheral B cells from patients post *S. aureus* infection using a monoclonal antibody discovery technology which conserves the cognate pairing of antibody heavy and light chains [34]. Both plasma and memory B-cells were used as genetic source for the recombinant full length IgG repertoires (manuscript in preparation). Individual antibody clones were expressed by transfection of mammalian cells [35]. Supernatants containing full length IgG1 antibodies were harvested after seven days and used to screen for antigen binding by ELISA. Antibodies 4675, SD2, SD3 and SD4 were positive for binding to cell wall preparations from USA300 or Newman *S. aureus* strains. Antibodies were subsequently produced in 200-ml transient transfections and purified with Protein A chromatography (MabSelect SuRe, GE Life Sciences, Piscataway, NJ) for further testing. Isolation and usage of these antibodies were approved by the regional ethical review board.

Mouse mAb against ClfA (9E10), ClfB, (10D2), SdrD (17H4), IsdA (2D3) and non-modified SDR proteins (9G4) were generated by immunizing mice with the respective recombinant proteins, which were purified after expression in *E. coli*, using standard protocols; hybridoma supernatants were purified by protein A affinity chromotography. Rabbit mAb 28.9.9 was generated by immunizing rabbits with peptidoglycan (PGN)-derived peptide CKKGGG-(L-Ala)-(D-gamma-Glu)-(L-Lys)-(D-Ala)-D-Ala) followed by cloning of the IgG.

### Bacterial cell wall preparations (CWP), immunoblotting, and ELISA

CWP were generated by incubating 40 mg of pelleted *S. aureus* or *S. epidermidis* per mL of 10 mM Tris-HCl (pH 7.4) supplemented with 30% raffinose, 100 µg/ml of lysostaphin (Cell Sciences, Canton, MA), and EDTA-free protease inhibitor cocktail (Roche, Pleasanton, CA), for 30 min at 37°C. The lysates were centrifuged at 11,600 x g for 5 min, and the supernatants containing cell wall components were collected. For immunoprecipitation, CWP were diluted 4 times in NP-40 buffer (120 mM NaCl, 50 mM Tris-HCl pH 8.0, 1% NP-40, complete protease inhibitor cocktail (Roche) and 2 mM dithiothreitol) containing 1 µg/mL of indicated primary antibodies and incubated for 2 h at 4°C, followed by a 1 h incubation with Protein A/G agarose (Thermo, Waltham, MA). Whole cell lysates (WCL) were generated by a 30 min incubation at 37°C in 20 mM Tris-HCl (pH 7.4), 150 mM NaCl, 100 µg/ml of lysostaphin, 1% Triton-X100 (Thermo) and EDTA-free protease inhibitor cocktail. For immunoblot analysis, proteins were separated on a 4-12% Tris-glycine gel, and transferred to a nitrocellulose membrane (Invitrogen, Carlsbad, CA), followed by blotting with indicated primary antibodies (1 µg/mL). Antibodies used are listed in Table 1. Lectin studies were performed by immunoprecipitating filtered (0.2 micron) overnight culture supernatants with concanavalin A (ConA)- or sWGA-agarose beads (Vector Labs, Burlingame, CA) supplemented with 0.1 mM CaCl_2_ and 0.01 mM MnCl_2_.

ELISA experiments were performed using standard protocols. Briefly, plates which were pre-coated with CWP were reacted with human IgG preparations, ie. purified human IgG (Sigma), intravenous immunoglobulin Gammagard Liquid (Baxter, Westlake Village, CA), pooled serum from healthy donors or from MRSA patients (both generated in-house). The concentrations of anti-staphylococcal IgG present in the serum or purified IgG were calculated by using a calibration curve that was generated with known concentrations of mAb 28.9.9 against peptidoglycan.

### Expression of MBP-fusion proteins in various bacterial strains

For inducible expression in *S. aureus* or *B. subtilis*, constructs of maltose binding protein (MBP) were fused to various PCR-amplified clumping factor A (CflA) domains (listed in Table 1) and cloned into vector pTet.MK4, which was modified from pMK4 (ATCC). The pTet.MK4 constructs were electroporated into Δ*mcr* USA300 NRS384 (including SdgA/B mutant strains), Δ*spa* RN4220, or *B. subtilis*. Protein expression was induced by culture in TSB for 2.5 h at 37°C with 200 ng/ml of anhydrotetracycline (Sigma). For inducible expression in *E. coli*, constructs were cloned into expression vector pMal.c5x (New England Biolabs, Ipswich, MA), and expression was induced by culture in the presence of 0.3 mM IPTG (Sigma). The cultures were resuspended in lysis buffer (1% TritonX-100, 150 mM NaCl, 20 mM Tris pH 7.5, EDTA-free protease inhibitor cocktail [Roche]), and bacteria were lysed by incubation at 37°C for 30 minutes with either 100 µg/ml of lysostaphin (*S. aureus*), or with 6 kU/ml of lysozyme (*B. subtilis and E.coli*), followed by mechanical disruption using a Mini-Beadbeater (Biospec Products, Bartlesville, OK). The lysates were centrifuged at 18,000 x g for 10 minutes, and the supernatants were incubated with Ni-Nta resin (Qiagen, Valencia, CA) for 1.5 h at 4°C or with amylose agarose resin (New England Biolabs) for 1.5 hrs at 4°C. The resins were washed 3 times with PBS containing 10 mM imidazole and 1% NP40, and samples were analyzed by immunoblotting.

### *In vitro* glycosylation of SDR proteins expressed in *E.coli*

The *clfA*, *clfB*, *sdrC*, *sdrD* and *sdrE* genes were PCR amplified from the mature start of the protein to the glycine in the LPXTG motif from USA300 genomic DNA and ligated in frame with an NT Unizyme tag. The constructs were transformed into *E. coli* for protein expression. Proteins were purified from *E. coli* lysates using a Ni-NTA resin (Qiagen) in PBS with protease inhibitors (Roche) for 1.5 h at 4 °C. These resin-bound SDR-proteins were subjected to *in vitro* glycosylation by *S. aureus* lysates as follows. First, Δ*panSDR* mutant *S. aureus* from a log-phase culture were lysed by incubation in PBS containing 200 ug/ml lysostaphin and 250 U/ml of benozase nuclease (Novagen, Madison, WI) for 30 min at 37°C, followed by centrifugation at 18,000 x g to remove debris. Next, *in vitro* glycosyl modification of resin-bound *E. coli* SDR-proteins was induced by addition of Δ*panSDR* mutant *S. aureus* lysates and incubation for 1 h at 37°C, followed by 3 washes with a buffer containing 50 mM NaH_2_PO_4_, 300 mM NaCl, 10 mM imidazole (pH 8.0).

For *in vitro* glycosylation by purified glycosyltransferases, the full lengths of the indicated *S. aureus* glycosyltransferase genes were PCR amplified and ligated in frame with an N-terminal 6x His-tag into the pET15b *E. coli* expression vector, and transformed into *E. coli*, followed by protein expression and purification. MBP-SDR709 fusion protein was cloned into pMal.c5x, and expressed and purified as described above. Purified MBP-SDR709 fusion protein was incubated with amylose agarose (New England Biolabs) for 1.5 h at 4°C. To provide necessary building sugar blocks, a *B. subtilis* lysate was prepared by resuspension of a logarithmic culture of *B. subtilis* in PBS with protease inhibitors (Roche) and mechanical disruption using a Mini-Beadbeater, followed by removal of debris by centrifugation. To induce glycosylation, the amylose captured MBP-SDR709 fusion protein was incubated with the *B. subtilis* lysate in the presence of 10 µg/ml of purified glycosyltransferase for 1 h at 37°C, followed by 3 washes in 1% NP-40 buffer. Binding of glycosyltransferases to MBP-SDR709 fusion protein was assessed as follows. Two µg of purified MBP-SDR709 protein was incubated with 10 µg of a purified His-tagged glycosyltransferase in 1% NP-40 buffer and protease inhibitors for 2 h at 4°C. This was followed by incubation with amylose agarose for 1 h at 4°C, and 3 washes with 1% NP-40 buffer.

For co-expression of SDR protein with Sdg enzymes in *E. coli*, a NT-His tag fused to the full length of either SdgA or SdgB was cloned into the arabinose inducible expression vector pBad33 (ATCC), and transformed into the *E.coli* strain harboring the inducible MBP-SDR709 fusion protein (expressed in the pMal.c5x expression vector; see above). Expression of both SDR protein and Sdg enzyme was induced by addition of 0.2% L-(+)arabinose (Sigma) and 0.3 mM IPTG. Whole cell lysates were prepared by incubation in 100 mM Tris (pH 7.5) with 4% SDS, 1 mM EDTA, and 100 mM DTT for 5 min at 100°C.

For cell-free reconstitution of SDR-protein glycosylation, 100 µg of purified MBP-SDR709 protein, purified after expression in *E. coli*, was mixed with 30 µg of UDP-GlcNAc (Sigma) and 4 µg of purified SdgA and/or SdgB enzyme in 100 mM Tris. This mixture was incubated for 1 h at 37°C for 1 h, and subsequently analyzed by immunoblotting or mass spec. All *in vitro* glycosylated SDR-proteins were analyzed by immunoblotting as described above.

### Mass spectrometry analysis for the identification of glycosyltransferases and sugar moieties

For the identification of the SDR-modifying glycosyltransferases, lysates of Δ*panSDR* mutant *S. aureus* were prepared as described above and fractionated by sequential size exclusion and anion exchange chromatography. Aliquots of fractions were separated on a 4–20% Tris-glycine gel, and fractions which stained positive with rF1 by immunoblotting were again separated by SDS-PAGE, subjected to in-gel trypsin (Promega, Madison, WI) digestion, followed by mass spectrometric analysis as previously described [36]. Tandem mass spectra were submitted for database searching using the Mascot program version 2.2.06 (Matrix Science) against a concatenated NCBInr target-decoy database consisting of staphylococcus aureus proteins and common laboratory contaminants. Peptide and protein identifications were validated with the Scaffold program version 2.06.01 (Proteome Software). Thresholds for accepting peptide and protein identifications were set at greater than 90% and 99% respectively, using the Peptide Prophet and Protein Prophet algorithms [37].

For the identification of the sugar moieties on SDR proteins, the MBP-SDR-His protein was expressed in *E. coli* and purified on a dextrin affinity column. Protein was analyzed by LC-MS using a PLRP-C (Agilent, Santa Clara, CA) reversed phase column connected to the electrospray orifice of an Agilent 6520 TOF run in positive ion mode. Proteins were chromatographed in 0.05% trifluoroacetic acid buffer and eluted with a gradient of acetonitrile.

### FACS analysis of rF1 binding to whole bacteria from culture or infected tissues

Whole bacteria were harvested from TSA plates or TSB cultures and washed with HBSS without phenol red supplemented with 0.1% IgG free BSA (Sigma) and 10 mM Hepes, pH 7.4 (HB buffer) Bacteria (20×10^8^ CFU/mL) were incubated with 300 µg/mL of rabbit IgG (Sigma) in HB buffer for 1 h at room temperature (RT) to block nonspecific IgG binding. Bacteria were stained with 2 µg/mL of primary antibodies, including rF1 or isotype control IgG1 mAb mAb gD:5237 [38], and next with fluorescent anti-human IgG secondary antibodies (Jackson Immunoresearch, West Grove, PA). The bacteria were washed and analyzed by FACSCalibur (BD).

For antibody staining of bacteria from infected mouse tissues, 6–8 weeks old female C57Bl/6 mice (Charles River, Wilmington, MA) were injected intravenously with 10^8^ CFU of logphase-grown USA300 in PBS. Mouse organs were harvested two days after infection. Rabbit infective endocarditis (IE) was established as previously described [39]. Rabbits were injected intravenously with 5×10^7^ CFU of stationary-phase grown MRSA strain COL, and heart vegetations were harvested eighteen hours later. Treatment with 30 mg/kg of vancomycin was given intravenously b.i.d. 18 h after infection with 7×10^7^ CFU stationary-phase COL.

To lyse mouse or rabbit cells, tissues were homogenized in M tubes (Miltenyi, Auburn, CA) using a gentleMACS cell dissociator (Miltenyi), followed by incubation for 10 min at RT in PBS containing 0.1% Triton-X100 (Thermo), 10 µg/mL of DNAseI (Roche) and Complete Mini protease inhibitor cocktail (Roche). The suspensions were passed through a 40 micron filter (BD) and bacteria were stained with mAbs as described above. Bacteria were differentiated from mouse organ debris by double staining with 20 µg/mL mouse mAb 702 anti-*S. aureus* peptidoglycan (abcam, Cambridge, MA) and a fluorochrome-labeled anti-mouse IgG secondary antibody (Jackson Immunoresearch). During flow cytometry analysis, bacteria were gated for positive staining with mAb 702 from double fluorescence plots. All animal experiments were approved by the Institutional Review Boards of Genentech and the University of California, San Francisco.

### Killing of rF1-preopsonized MRSA by human PMN

Peripheral leukocytes were enriched for polymorhonuclear cells (PMN) by dextran sedimentation. Briefly, equal volumes of healthy donor peripheral blood collected in EDTA tubes were mixed with a 0.9% NaCl solution containing 3% 500 kD dextran (Sigma), and left to sediment for 20 min at RT. The leukocytes in the upper layer were washed twice with HB buffer and resuspended at 3×1
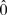
6/mL. USA300 bacteria were washed, resuspended at 30×1
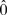
6/mL in HB, and preopsonized for 30 min at 37°C with rF1, human mAb 4675 anti-ClfA, or human IgG1 control mAb gD:5237. Bacteria were spun down at 2500 rpm for 5 min, and PMN were added at a bacteria:PMN ratio of 10∶1. After incubation for 90 min at 37C, the suspensions were diluted 10-fold in water and left for 1 min at RT to lyze the PMN. Serial 10-fold dilutions in PBS containing 0.05% Tween-20 were cultured on TSA plates to determine numbers of viable CFU.

### Treatment of bacteria with human neutrophil proteases or lysosomal extracts from human neutrophils and cultured cells

Lysosomal extracts were isolated from human neutrophils, THP-1 cells, and RAW cells, using a Lysosome Enrichment kit (Thermo). A total of 5×10^7^ cells was used to obtain 300 to 500 microgram of total proteins in the lysosomes. Protease inhibitors were omitted from all steps to maintain protease activity in the lysosomes. The plasma membranes of the cells were disrupted by 30 strokes using a dounce homogenizer (Wheaton, Millville, NJ). The homogenate was centrifuged at 500 x g for 5 min to obtain postnuclear supernatant, which was loaded onto the top of a gradient of 8%, 20%, 23%, 27% and 30% (from top to bottom) of iodixanol. After ultracentrifugation at 145,000 x g for 2 h at 4°C, we obtained the lysosomes layered between 8% and 20% iodixanol. This lysosomal fraction was diluted into PBS and pelleted by centrifugation at 18,000 x g for 30 min at 4°C. The lysosomal pellets were washed with PBS and lysed in 2% CHAPS with Tris-buffered saline to obtain lysosomal extracts.

To analyze the cleavage of SDR proteins by host proteases, *S. aureus* bacteria were treated with 50 nM of purified human neutrophil serine proteases or 0.1 mg/ml of neutrophil lysosomal extracts in 50 mM Tris (pH 8.0) with 150 mM NaCl and 2 mM CaCl_2_; or with 0.1 mg/ml of RAW or THP-1 lysosomal extracts in 50 mM NaCitrate with 100 mM NaCl and 2 mM DTT (pH 5.5). Cathepsin G inhibitor (Calbiochem, Billerica, MA) was added at 100 µg/ml. These mixtures were incubated at 37°C for 30 minutes when using purified proteases or for 1 h when using lysosomal lysates, and centrifuged to pellet bacteria. The supernatants were analyzed by immunoblotting to detect cleavage products. In some experiments, cell wall preparations were obtained from the remaining bacterial pellets and also analyzed by immunoblotting.

### Adherence of bacteria to human fibrinogen

Bacteria from 4 h logphase culture in TSB were washed by centrifugation and resuspended at a concentration of 3×10^8^/mL in 50 mM TrisHCl (pH 8.0) with 150 mM NaCl and 2 mM CaCl_2_. Human cathepsin G (Athens, Athens, GA) was added at a variety of concentrations. The bacteria were incubated for 30 min at 37°C, followed by one centrifugation and resuspension at 3×10^8^/mL in PBS with 2% IgG-free BSA (Sigma). These suspensions were transferred to 2HB Immunon 96-well plates (Thermo) which were precoated with human fibrinogen (Fluka/Sigma) at 20 µg/mL in carbonate buffer (pH 9.0). The plates were gently rocked for 1 h at room temperature, and washed three times using 10 mM Tris (pH 8.0) containing 150 mM NaCl and 0.1% Tween-20. To quantify the number of bacteria remaining adherent to the plate-bound fibrinogen, the amount of bacterial ATP was measured using the BacTiterGlo kit (Promega), and fluorescence was expressed in arbitrary units (AU) as a measure for bacterial adherence.

## Supporting Information

Figure S1 mAb rF1 binds to all 15 S. aureus strains tested (related to Figure 1E). Various methicillin-resistant *S. aureus* (MRSA), vancomycin intermediate resistant *S. aureus* (VISA), and methicillin-sensitive *S. aureus* (MSSA) strains were incubated with mAb rF1 (red lines), and as controls with isotype-matched IgG1 mAb anti-gD (blue lines), or without mAb (green lines). Binding of mAb to *S. aureus* bacteria was determined by flow cytometry. Note that for some strains (N315 and Newman), the background staining with isotype control is slightly higher, presumably because of higher expression of IgG-binding protein A. All strains are listed in Table 1.
(PDF)

Figure S2 (related to Figure 2C). Protein sequence coverage map of SDR-proteins identified by mass spectrometry from rF1-immunoprecipitates of *S. epidermidis* lysates. Residues highlighted in yellow indicate portion of sequence detected, oxidized methionine highlighted in green. (A) Sdr F, (B) Sdr G, (C) Sdr H.
(PDF)

Figure S3 (related to Figure 3C). Protein sequence coverage map of glycosyltransferase family proteins identified by mass spectrometry from Δ*panSDR* mutant *S. aureus* lysate fractionated by size exclusion, followed by anion exchange chromatography. Residues highlighted in yellow indicate portion of sequence detected, oxidized methionine highlighted in green. (A) SdgA, (B) SdgB, (C) TarM, (D) TarS (SAUSA300_0252).
(PDF)
