# Supplementary material for: Novel Staphylococcal Glycosyltransferases SdgA and SdgB Mediate Immunogenicity and Protection of Virulence-Associated Cell Wall Proteins
Source: PLoS Pathog. 2013 Oct 10;9(10):e1003653. doi: 10.1371/journal.ppat.1003653 (PMC3794999; doi:10.1371/journal.ppat.1003653)
Supplement: Figure S1 — mAb rF1 binds to all 15 S. aureus strains tested (related to Figure 1E). Various methicillin-resistant S. aureus (MRSA), vancomycin intermediate resistant S. aureus (VISA), and methicillin-sensitive S. aureus (MSSA) strains were incubated with mAb rF1 (red lines), and as controls with isotype-matched IgG1 mAb anti-gD (blue lines), or without mAb (green lines). Binding of mAb to S. aureus bacteria was determined by flow cytometry. Note that for some strains (N315 and Newman), the background staining with isotype control is slightly higher, presumably because of higher expression of IgG-binding protein A. All strains are listed in Table 1. (PDF) [file ppat.1003653.s001.pdf]

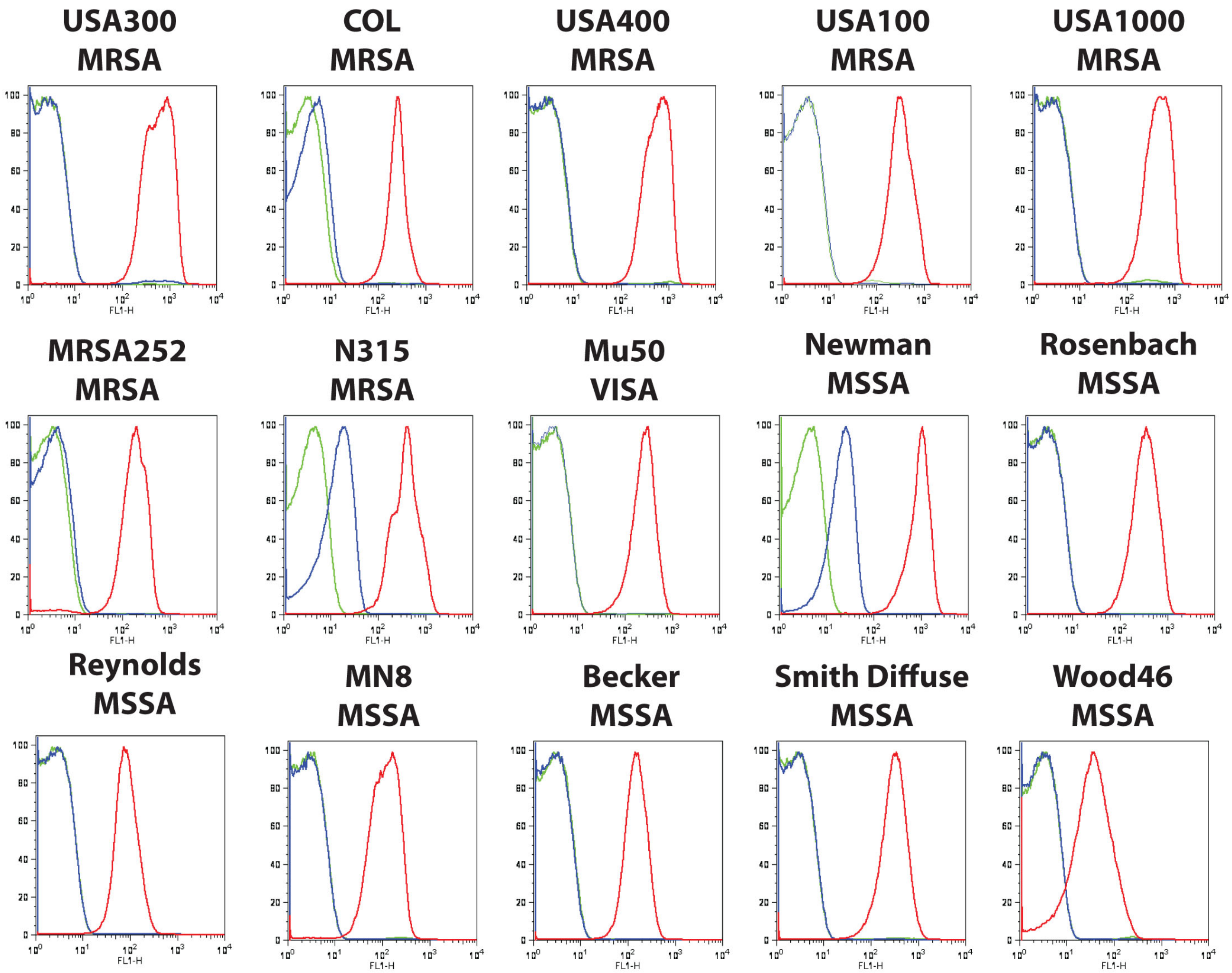

Supporting Figure S1. mAb rF1 binds to all 15 *S. aureus* strains tested (related to Figure 1E). Various methicillin-resistant *S. aureus* (MRSA), vancomycin intermediate resistant *S. aureus* (VISA), and methicillin-sensitive *S. aureus* (MSSA) strains were incubated with mAb rF1 (red lines), and as controls with isotype-matched IgG1 mAb anti-gD (blue lines), or without mAb (green lines). Binding of mAb to *S. aureus* bacteria was determined by flow cytometry. Note that for some strains (N315 and Newman), the background staining with isotype control is slightly higher, presumably because of higher expression of IgG-binding protein A. All strains are listed in Table 1.
