# Supplementary material for: Novel Staphylococcal Glycosyltransferases SdgA and SdgB Mediate Immunogenicity and Protection of Virulence-Associated Cell Wall Proteins
Source: PLoS Pathog. 2013 Oct 10;9(10):e1003653. doi: 10.1371/journal.ppat.1003653 (PMC3794999; doi:10.1371/journal.ppat.1003653)
Supplement: Figure S2 — (related to Figure 2C). Protein sequence coverage map of SDR-proteins identified by mass spectrometry from rF1-immunoprecipitates of S. epidermidis lysates. Residues highlighted in yellow indicate portion of sequence detected, oxidized methionine highlighted in green. (A) Sdr F, (B) Sdr G, (C) Sdr H. (PDF) [file ppat.1003653.s002.pdf]

A

MKKRRQGPIN KRVDFLSNKV NKYSIRKFTV GTASILVGAT LMFGAADNEA KAAEDNQLES
ASKEEQKGSR DNESSKLNQV DLDNGSHSSE KTTNVNNATE VKKVEAPTTS DVSKPKANEA
VVTNESTKPK TTEAPTVNEE SIAETPKTST TQQDSTEKNN PSLKDNLNSS STTSKESKTD
EHSTKQAQMS TNKSNLDTND SPTQSEKTSS QANNDSTDNQ SAPSKQLDSK PSEQKVYKTK
FNDEPTQDVE HTTTKLKTPS ISTDSSVNDK QDYTRSAVAS LGVDSNETEA ITNAVRDNLD
LKAASREQIN EAIIAEALKK **DFSNPDYGVD** **TPLALNTSQS** **K**NSPHKSASP R**MNLMSLAAE**
PNSGKNVNDK VK**ITNPTLSL** **NKSNNHANNV** **IWPTSNEQFN** **LKANYELDDS** **IKEGDTFTIK**
YGQYIRPGGL **ELPAIK**TQLR SK**DGSIVANG** **VYDK**TTNTTT YTFTNYVDQY QNITGSFDLI
ATPK**RETAIK** **DNQNYPMEVT** **IANEVVKKDF** **IVDYGNKKDN** **TTTAAVANVD** **NVNNKHNEVV**
YLNQNNQNPK YAKYFSTVKN GKFIPGEVK**V** **YEVTDTNAMV** **DSFNPDLNSS** **NVKDVTSQFT**
PKVSADGTR**V** **DINFAR**SMAN GKK**YIVTQAV** **RPTGTGNVYT** **EYWLTRDGTT** **NTNDFYR**GTK
STTVTYLNGS STAQGDNPTY SLGDYVWLDK NK**NGVQDDDE** **KGLAGVYVTL** **K**DSNNRELQR
VTTDQSGHYQ FDNLQNGTYT VEFAIPDNYT PSPANNSTND AIDSDGERDG TRKVVVAK**GT**
INNADNMTVD **TGFYLTPKYN** **VGDYVWEDTN** **KDGIQDDNEK** GISNVKVTLK NKNGDTIGTT
TTDSNGKYEF TGLENGDYTI EFETPEGYTP TKQNSGSDEG KDSNGTKTTV TVKDADNK**TI**
DSGFYKPIYN **LGDYVWEDTN** **KDGIQDDSEK** GISGVKVTLK DK**NGNAIGTT** **TTDASGHYQF**
KGLENGSYTV **EFETPSGYTP** **TK**ANSGQDIT VDSNGITTTG IINGADNLTI DSGFYKTPK**Y**
SVGDYVWEDT **NKDGIQDDNE** **K**GISGVK**VTL** **KDEK**GNIIST TTTDENGKYQ FDNLDSGNYI
IHFEKPEGMT QTTANSGNDD EKDADGEDVR VTITDHDDFS IDNGYFDDDS DSDSDADSDS
DSDSDSDADS DSDADSDSDS DSDSDSDSDS DSDSDSDSDS DSDSDADSDS DADSDSDSDS
DSDSDSDSDS DSDSDSDSDS DSDADSDSDA DSDSDADSDS DADSDSDSDS DSDADSDSDS
DSDSDADSDS DSDSDSDADS DSDSDSDSDA DSDSDSDSDS DADSDSDSDS DSDSDSDSDS
DSDSDSDSDS DSDSDSDSDS DSDSDADSDS DSDSDSDADS DSDADSDSDS DSDSDSDSDS
DADSDSDSDS DSDSDSDSDA DSDSDSDSDS DAESDSDSDS DSDSDSDSDS DSDSDSDSDS
DSDSDSDADS DSDSDSDSDS DSDSDSDSDS DSDSDSDADS DSYSDSDSDS DSDSDSDSDS
DSDSDSDSDS DSDSDSDSDS DSDSDSDSDS DSDSDSDSDS DSDSDSDSDS DSDSDSDSDS
DSDSDSDSDS DSDSDSDSDS DSDSDSDKNA KDKLPDTGAN EDHDSKGTLL GTLFAGLGAL
LLGRRRKKDN KEK

B

MFGLGHNEAK AEENSVQDVK DSNTDDELSD SNDQSSDEEK NDVINNNQSI NTDDNNQIIK
KEETNNYDGI EKRSEDRTES TTNVDENEAT FLQKTLQDNT HLTEEEVKES SSVESSNSSI
DTAQQPSHTT INREESVQTS DNVEDSHVSD FANSKIKESN TESGKEENTI EQPNK**VKEDS**
TTSQPSGYTN **IDEKISNQDE** **LLNLPINEYE** **NKARPLSTTS** **AQPSIKRVTV** **NQLAAEQGSN**
VNHLIKVTDQ **SITEGYDDSE** **GVIKAHDAEN** **LIYDVTFEVD** **DKVKSGDTMT** **VDIDKNTVPS**
DLTDSFTIPK **IKDNSGEIIA** **TGTYDNK**NK**Q** **ITYTFTDYVD** **KYENIK**AHLK **LTSYIDK**SKV
PNNNTKLDVE YK**TALSSVNK** TITVEYQRPN ENR**TANLQSM** **FTNIDTKNHT** **VEQTIYINPL**
RYSAK**ETNVN** **ISGNGDEGST** **IIDDSTIIK**V YK**VGDNQNLP** **DSNRIYDYSE** **YEDVTNDDYA**
QLGNNNDVNI **NFGNIDSPYI** **IK**VISK**YDPN** **KDDYTTIQQT** **VTMQTTINEY** **TGEFRTASYD**
NTIAFSTSSG **QGQGDLPPEK** TYKIGDYVWE DVDKDGIQNT NDNEKPLSNV LVTLTYPDGT
SKSVR**TDEDG** **KYQFDGLK**NG LTYK**ITFETP** **EGYTPTLK**HS GTNPALDSEG NSVWVTINGQ
DDMTIDSGFY QTPK**YSLGNY** **VWYDTNKDGI** **QGDDEK**GISG VK**VTLKDENG** **NIISTTTTDE**
NGKYQFDNLN SGNYIVHFDK PSGMTQTTTD SGDDDEQDAD GEEVHVTITD HDDFSIDNGY
YDDESDSDSD SDSDSDSDSD SDSDSDSDSD SDSDSDSDSD SDSDSDSDSD SDSDSDSDSD
SDSDSDSDSD SDSDSDSDSD SDSD

C

MKKFNIKHSF MLTGFAFMVT TSLFSHQAHA EGNHPIDINF SKDQIDRNTA KSNIINRAND
TSRTGISMNS DNDLDTDIVS NSDSENDTYL DSDSDSDSDL DSDSDSDSDS DSDSDSDSDS
DSDSDSDSDS DSDSDSDSDS DSDSDSDSDS DSDSDSDSDS DSDSDSDSDS DSDSDSDSDS
DSDSDSDSDS DSDSDSDSGT SSGKGSHTGK KPGNPKGNTN RPSQRHTNQP QRPKYNQTNQ
NNINNINHNI NHTR**TSGDGA** **PFKR**QQNIIN SNSGHRNQNN INQFIWNKNG FFKSQNNTEH
R**MNSSDNTNS** **LISR**FRQLAT GAYKYNPFLI NQVKNLNQLD GK**VTDSDIYS** **LFR**KQSFRGN
EYLNSLQKGT SYFRFQYFNP LNSSKYYENL DDQVLALITG EIGSMPELKK PTDKEDKNHS
AFKNHSADEI TTNNDGHSKD YDKKKKIHRS LLSLSIAIIG IFLGVTGLYI FRRKK

Supporting Figure S2 (related to Figure 2C). Protein sequence coverage map of SDR-proteins identified by mass spectrometry from rF1-immunoprecipitates of *S. epidermidis* lysates. Residues highlighted in yellow indicate portion of sequence detected, oxidized methionine highlighted in green. (A) Sdr F, (B) Sdr G, (C) Sdr H.
