# Supplementary material for: Novel Staphylococcal Glycosyltransferases SdgA and SdgB Mediate Immunogenicity and Protection of Virulence-Associated Cell Wall Proteins
Source: PLoS Pathog. 2013 Oct 10;9(10):e1003653. doi: 10.1371/journal.ppat.1003653 (PMC3794999; doi:10.1371/journal.ppat.1003653)
Supplement: Figure S3 — (related to Figure 3C). Protein sequence coverage map of glycosyltransferase family proteins identified by mass spectrometry from ΔpanSDR mutant S. aureus lysate fractionated by size exclusion, followed by anion exchange chromatography. Residues highlighted in yellow indicate portion of sequence detected, oxidized methionine highlighted in green. (A) SdgA, (B) SdgB, (C) TarM, (D) TarS (SAUSA300_0252). (PDF) [file ppat.1003653.s003.pdf]

A

MNYFVGNSLG VNLTGIEKAI INRLNLFKEM GRPAQCVFLS WNRYLYRNAQ NYITSSDYIN
MYDFFQEATY LERNEPFDWL SYWTDECHYT LKHVENSHDF RIYDQERFLM YAHFQDPKYR
ILDYVNHFDS **QR**RKVKRDFY DVRGFLSCSR ILVDKQQTLC EFFYNPEGDT KLEKYFSYKD
GKPEVQKIIV YYANKQYFFN NETELGAFFI KQLYQHGDLF FSDRNVYTAP IFNLTPESIP
VVAVLHSTHI K**NIDALDSSP** **FK**NVYKAMFE NLSRYR**AIIV** **STEQQK**LDIE KR**INHTIPVV**
NIPVGYSETI **DTPVQTLDQR** SVKLISVARY SPEKQLHQQI ELIKRLVSYV PKIELHMYGF
GSESKKLNEL IQK**YGLENHV** **YLR**GFLSNLD QEYSDAYLSL ITSNMEGFSL ALLESLAHGV
PVISYDIKYG PNELITPDFN GYLITKNDED ALFDKVK**YVI** **DHPEVQQR**LS KGSLAKAQQY
SKASLIKQWD QFVRLI

B

MNYILGTILE SKITGVEKAQ INRLKLFKQH GISSKCVYVK WNPYSYTYAK QHQIENDVFT
MYDYFQKAIN YKKTKQVNWI QYWEKSCRYT LK**FVENSNDV** **R**IYDEEQFVM YAHFLDKQYH
QLNYVNYFDH KRRKVKRELY DGRGFLSCSR ILGEGQRIVL ENYYTPNGEI VIQKYFDDIK
GKNTLTKVIL NEDQQQQFFD TEDELVQYFL HQLCKNNDQI ILDRPHELGN VIAGLNQSIP
VVVVLHSTHL SGTGNGIKSF YKTVFNNLTR YKAIVVSTEQ QCQDISQYIE NK**IPVINIPV**
GYVANLKYQF **DINQK**EKNHI ISIARLVENK QIK**HQIEVIK** QLVTKHPNIQ LNIYGHGNGL
SEYRQLVEDY HLSEHVKFHG FK**THINEEIA** **K**AELMLSTSK MEGFGLAILE SLSVGTPVIS
YDIDYGPSEL IQDGFNGYLV PKGDINQMVE K**VDQLLNNTQ** **K**LQQFSINSI ESAQQYNATT
ISTKWQNILN

C

MKK**IFMMVHE** **LDVNKGGMTS** **SMFNR**SK**EFY** **DADIPADIVT** **FDYK**GNYDEI IKALKKQGKM
DRRTK**MYNVF** **EYFK**QISNNK HFKSNKLLYK HISER**LKNTI** **EIEESK**GISR YFDITTGTYI
AYIRKSKSEK VIDFFKDNKR IERFSFIDNK VHMKETFNVD NKVCYQVFYD EKGYPYISRN
INANNGAVGK **TYVLVNKK**EF KNNLALCVYY LEKLIKDSKD SIMICDGPGS FPKMFNTNHK
NAQKYGVIHV NHHENFDDTG AFKKSEKYII ENANK**INGVI** **VLTEAQR**LDI LNQFDVENIF
TISNFVKIHN APKHFQTEKI VGHISRMVPT KR**IDLLIEVA** **ELVVK**KDNAV K**FHIYGEGSV**
KDKIAKMIED KNLERNVFLK GYTTTPQKCL EDFKLVVSTS QYEGQGLSMI EAMISKRPVV
AFDIK**YGPSD** **FIEDNK**NGYL IENHNIKDMA DK**ILQLVNND** **VLAAEFGSK**A RENIIEKYST
ESILEKWLNL FNS

D

MK**FSVIVPTY** **NSEKYITELL** **NSLAK**QDFPK TEFEVVVVDD CSTDQTLQIV EKYRNKLNLK
VSQLETNSGG **PGKPR**NVALK **QAEGEFVLFV** **DSDDYINK**ET LK**DAAAFIDE** **HHSDVLLIK**M
KGVNGRGVPQ SMFK**ETAPEV** **TLLNSRIIYT** **LSPTK**IYRTA LLK**DNDIYFP** **EELKSAEDQL**
FTMKAYLNAN **R**ISVLSDKAY YYATKR**EGEH** **MSSAYVSPED** **FYEVMRLIAV** **EILNTDLEEA**
HKNQILAEFL **NRHFSFSR**TN GFSLKVKLED QPQWINALGD FIQAVPER**VD** **ALVMSKLRPL**
LHYARAKDID **NYR**TVEESYR QGQYYRFDIV DGK**LNIQFNE** **GEPYFKGIDI** **AKPK**VKMTAF
KFDNHK**IVTE** **LTLNEFMIGE** **GHYDVR**LKLH SRNKK**HTMYV** **PLSVNANK**QY R**FNIMLEDIK**
AYLPKEKIWD VFLEVQIGTE VFEVRVGNQR NK**YAYTAETS** **ALIHLNNDFY** **R**LTPYFTKDF
NNISLYFTAI TLTDSISLKL KGKNK**IILTG** **LDR**GYVFEEG MASVVLKDDM VMGMLSQTSE
NEVQILLSKD IKKRDFKNIV K**LNTAHITYP** **LNK**

Supporting Figure S3 (related to Figure 3C). Protein sequence coverage map of glycosyltransferase family proteins identified by mass spectrometry from Δ*panSDR* mutant *S. aureus* lysate fractionated by size exclusion, followed by anion exchange chromatography. Residues highlighted in yellow indicate portion of sequence detected, oxidized methionine highlighted in green. (A) SdgA, (B) SdgB, (C) TarM, (D) TarS (USA300_0252).
